# System-, teacher-, and student-level interventions for improving participation in online learning at scale in high schools

**DOI:** 10.1073/pnas.2216686120

**Published:** 2023-07-17

**Authors:** Igor Asanov, Anastasiya-Mariya Asanov, Thomas Åstebro, Guido Buenstorf, Bruno Crépon, David McKenzie, Francisco Pablo Flores T., Mona Mensmann, Mathis Schulte

**Affiliations:** ^a^International Center for Higher Education Research, University of Kassel, 34125 Kassel, Germany; ^b^Economics and Decision Sciences, HEC Paris, 78350 Jouy-en-Josas, France; ^c^Department of Economics, Ecole Nationale de la Statistique, 91120 Palaiseau, France; ^d^Department of Economics, École Polytechnique, 91120 Palaiseau, France; ^e^Development Research Group, World Bank, Washington, DC 20433; ^f^Faculty of Management, Economics and Social Sciences (WiSo-Faculty), University of Cologne, 50923 Köln, Germany

**Keywords:** online education, nudge interventions, scale experiments, centralized monitoring

## Abstract

Many school systems across the globe turned to online education during the COVID-19 pandemic. This context differs significantly from the prepandemic situation in which massive open online courses attracted large numbers of voluntary learners who struggled with completion. Students who are provided online courses by their high schools also have their behavior determined by actions of their teachers and school system. We conducted experiments to improve participation in online learning before, during, and right after the COVID-19 outbreak, with 1,151 schools covering more than 45,000 students in their final years of high school in Ecuador. These experiments tested light-touch interventions at scale, motivated by behavioral science, and were carried out at three levels: that of the system, teacher, and student. We find the largest impacts come from intervening at the system level. A cheap, online learning management system for centralized monitoring increased participation by 0.21 SD and subject knowledge by 0.13 SD relative to decentralized management. Centralized management is particularly effective for underperforming schools. Teacher-level nudges in the form of benchmarking emails, encouragement messages, and administrative reminders did not improve student participation. There was no significant impact of encouragement messages to students, or in having them plan and team-up with peers. Small financial incentives in the form of lottery prizes for finishing lessons did increase study time, but was less cost-effective, and had no significant impact on knowledge. The results show the difficulty in incentivizing online learning at scale, and a key role for central monitoring.

## Significance

The COVID-19 pandemic saw many school systems turn to online education. In contrast to the voluntary learners in massive open online courses, school student engagement in online learning is also determined by the role of their teachers and schools. Using large-scale experiments with over 45,000 Ecuadorian high school students, we implemented rapid behavioral science interventions at the student, teacher, and system level. The largest impacts on study time and knowledge are seen from using centralized management over schools, rather than decentralized self-management. Teacher-level interventions had limited impact. Small financial incentives induce more student studying, but do not translate into more knowledge. Our findings highlight the need for moving beyond the student level in online education interventions.

The COVID-19 pandemic closed schools around the world, affecting more than 1.6 billion students United Nations Educational, Scientific and Cultural Organization (UNESCO, 2020). In response, more than 90 percent of countries implemented some form of remote learning policy, with digital instruction the most common method of instruction, particularly in the upper levels of high school (1).^*^ One common approach used was to provide self-paced online content that students could work through on their own. This may be particularly important in areas where internet bandwidth, student schedules, and teacher expertise limited the adoption of live Zoom-based classes, with students preferring to use mobile devices to work through interactive online lessons. However, a key concern then raised was how to ensure sufficient student participation in this online learning (2).

One potential source for guiding efforts to increase participation in online learning at scale comes from the experience of massive open online courses (MOOCs). Course completion rates for MOOCs have generally been very low, with many courses reporting that fewer than 20 or even 10 percent of those who start the courses continuing to completion (3–5). In response, a range of student-level interventions in the form of reminders, reward badges, gamification, and other behavioral nudges have been tried on platforms such as Coursera, edX, and Khan Academy (5, 6). Such interventions offer insights for helping to engage students, but there are at least two important differences between the experience of MOOCs and online learning as part of a formal high school education system. The first is that most MOOCs involve voluntary learners, whereas the online education offered during COVID-19 was instead part of compulsory schooling for many students. We should therefore expect higher completion rates and greater incentives to complete the courses. Second, because this content is offered through a formal education system, student participation may also be affected by the actions of their assigned teachers, and through system-level management by the Education Ministry.

We use the rollout of interactive online course modules to over 45,000 Ecuadorian high school students in 1,151 schools before, and during, the COVID-19 pandemic to test how student-, teacher-, and system-level interventions can best be used to increase student participation in online learning. This enables testing of light-touch interventions that could be delivered rapidly at scale, deriving lessons for the design and implementation of online education as part of a formal high school education system.

## Interventions

We developed online course modules in entrepreneurial education, statistics and scientific thinking, and Spanish and English language that were designed for students in their final years of high school in Ecuador. Collaborating with the Ministry of Education in Ecuador, these materials were first offered through computer labs in schools beginning in September 2019, and then when the COVID-19 pandemic closed schools, were offered remotely for students to access from their homes. A rapid response survey found that three-quarters of students had access to internet from their homes, and our intervention focuses on this sample for whom technology is not the main binding constraint to learning (7). A key concern was ensuring that students would actively participate and progress through these lessons, and we implemented a series of experiments to test different interventions designed to increase time spent by students using the online learning platform. These interventions were based on prior research in economics, education, and behavioral science, with a key consideration being what was feasible to implement quickly and at scale.

All of our student-level interventions took place when students were asked to access the material remotely from their homes. An economic approach to encouraging participation in learning is to directly incentivize it through financial incentives (8–10). There is evidence that rewarding education inputs such as reading books or completion of learning modules may be more effective than rewarding outputs such as test scores (10, 11). Our first treatment uses these insights to provide a financial incentive for completing modules by providing students with a lottery ticket for monetary prizes each time they finished a lesson (*SI Appendix* provides more details on each intervention). There was a concern that this might cause students to rush through each module, so in a second area of the country, we experimented by providing a lottery ticket for lesson completion and for scoring well on tasks within the lessons.

Our other student-level interventions were designed to help students overcome internal constraints to learning such as issues of motivation and self-control. One treatment provided students with encouragement messages (12) on the screen aiming to convince them that they could finish the course despite the pandemic. A second treatment aimed to induce self-regulated learning by asking students to form plans of how they would study, and to share these plans with others in their household. This was motivated by behavioral science work showing how plan-making can increase completion in MOOCs (13), although later work found much weaker impacts at scale (5). Our final student-level intervention encouraged students to team up remotely with peers to work together to complete the program. This was based on the idea that peers may hold one another accountable (14).

Our first teacher intervention was the only one that took place during the pre-COVID period and was a benchmarking treatment. Treated teachers received a weekly email that showed the performance of their classes to those for all other classes of the same course type. This can be viewed as a form of a social comparison nudge (15), which could induce pressure on low-performing teachers to improve to conform with the social norm, although there is the risk that benchmarking may have negative impacts on high-performing teachers (16). Our other two teacher interventions occurred when students were accessing the online content from their homes during the pandemic. One treatment consisted of simple administrative SMS messages that reminded teachers that their classes were being monitored and instructing them to make sure they finished the program on time. We view this as a combination of reminder nudges (15) and of increasing the salience of monitoring without increasing the actual amount of monitoring. The other treatment consisted of sending teachers encouragement emails, which included a link to a video showcasing the experiences of students and teachers who had previously finished the course, and thanking them for their efforts to help their students complete the course. As well as acting to increase teacher motivation, this may serve as a form of social information nudge (15, 17) by highlighting that others have successfully completed the program.

Our final intervention takes place at the school system level. As part of the experience gained with waves 1 and 2, we developed a real-time online management system. Proponents of school-based management argue that decentralizing authority from the central government to the school level can improve the delivery of education (18), but it has also been argued that this autonomy may instead negatively impact student achievement in low-income countries (19). We randomize schools to either centralized management, in which Ministry of Education personnel have access to the management system and receive weekly take-up reports about each school, or to self-management, in which only teachers receive information from the management system about their class.

We run these experiments in three waves, covering schools in different geographical areas in Ecuador (*SI Appendix*, Fig. S2). *SI Appendix*, Table S9 shows that while average characteristics of schools, teachers, and students differ across these areas, there is considerable overlap in the distributions. *SI Appendix*, Table S10 shows our main results are robust to reweighting the data to balance observable characteristics.

## Results

We examine impacts on two primary outcomes that measure how much time students spend using the online learning platform, and how much they learn from doing so. Sustained usage of the online learning platform is much higher than in the typical MOOC, with the average student in our control group completing 23.6 of the assigned 27 lessons, and spending 1,750 min or just over 29 h using the platform. However, there is still room to improve, with between 55 percent (first wave) and 82 percent (second wave, when students were required to complete all modules to graduate) of students completing all modules. There was also considerable scope for students to improve how much they learned, with the median student in our control group getting only 22 to 44 percent of questions correct on subject knowledge tests.

The largest, and only statistically significant, impacts from our student-level interventions come from our lottery treatments. Table 1 shows that offering students an additional lottery ticket each time they finished a lesson resulted in a 0.083 SD (SE 0.028) increase in study time, and offering a lottery ticket that depended on both completing lessons and scoring well on platform tasks increased study time by 0.107 SD (SE 0.053). *SI Appendix*, Table S1 shows this increase in the study time index is largely driven by time spent on the platform, with an estimated effect of 76 min (SE 25) for the first lottery, and of 91 min more platform time (SE 43) for the second lottery. The impact of making a plan for study and sharing it with someone else in their household had no significant impact on study time (0.043 SD, SE 0.031), nor did teaming up remotely with a peer (0.072 SD, SE 0.047). However, we cannot reject in either case that the impact was equal to that of the lottery treatment (*P* = 0.145, *P* = 0.490). Providing students with encouragement messages on the platform also had no significant impact on time spent on the platform (0.036 SD, SE 0.028). Despite the increase in time spent on the platform from at least the lottery treatment, none of the student-level interventions had any significant improvement on knowledge scores, with point estimates of 0.025 SD or less. We do not detect any significant heterogeneity in the impact of these student-level interventions with respect to either student gender or socioeconomic status (*SI Appendix*, Table S2).

**Table 1.** Impact of at scale light-touch interventions on online learning participation and knowledge

	No. of students	Study time index	No. of students	Knowledge test index
Student-Level Interventions				
Lottery ticket for lesson completion	11,834	0.083***	11,680	0.007
		(0.028)		(0.024)
*P* value		0.003		0.753
q value		0.019		0.69
Encouragement messages on screen	11,834	0.036	11,680	0.025
		(0.028)		(0.024)
*P* value		0.204		0.282
q value		0.51		0.545
Plan and within household team-up	11,834	0.043	11,680	0.008
		(0.031)		(0.026)
*P* value		0.159		0.771
q value		0.516		0.69
Lottery ticket for lesson completion and score	12,422	0.107**	10,950	−0.064*
		(0.053)		(0.039)
*P* value		0.045		0.1
q value		0.191		0.191
Team-up with a peer	12,422	0.072	10,950	−0.04
		(0.047)		(0.036)
*P* value		0.12		0.271
q value		0.191		0.191
Teacher-Level Interventions				
Benchmarking emails	15,433	−0.023	12,298	0.025
		(0.071)		(0.033)
*P* value		0.745		0.435
q value		1		1
Administrative SMS	14,398	−0.076*	13,714	−0.016
		(0.044)		(0.042)
*P* value		0.088		0.71
q value		0.544		0.553
Encouragement emails.	14,398	−0.038	13714	0.053
		(0.046)		(0.039)
*P* value		0.405		0.178
q value		0.553		0.544
System-Level Intervention				
Centralized Monitoring: week 8	16,547	0.211*	8,318	0.108**
		(0.114)		(0.042)
*P* value		0.065		0.011
q value		0.053		0.047
Centralized Monitoring: week 11	16,547	0.023	14,562	0.126**
		(0.087)		(0.056)
*P* value		0.792		0.025
q value		0.247		0.047

Results presented are mean intention-to-treat effects, from regressions that include randomization strata dummies and baseline controls selected by postdouble selection lasso. SEs in parentheses are clustered at the level of randomization (e.g., class or school). *, **, and *** denote significance at the 10, 5, and 1 percent levels, respectively. To control the false-discovery rate, sharpened q values are calculated using the procedure in ref. 20, considering the experimental wave and level of intervention as a family. Study Time Index is an average of standardized z-scores of the number of minutes spent on the platform; number of active days; and number of lessons completed. Knowledge Test Index is an average of standardized z-scores for tests taken on the different subjects taught on the online platform.

None of the three teacher-level interventions significantly improved either study time or knowledge acquisition on average. The benchmarking treatment has a small negative average effect on study time of −0.023 SD (SE 0.071). We hypothesized that the impact of benchmarking may differ depending on whether it showed the teacher to be ahead of, or behind, the progress of other teachers. We find some evidence to support this hypothesis (*SI Appendix*, Table S3), with a significant interaction effect of 0.199 SD (*P* = 0.068) between benchmarking and having covered below the mean number of completed classes when the benchmarking intervention began. This implies a positive 0.07 SD impact for low-performing teachers, but a negative impact of −0.12 SD for initially high-performing teachers. Sending teachers administrative Short Message Service (SMS) messages reminding them that their activity is monitored and to complete the program on time had a negative impact of −0.076 SD (SE 0.047) on study time (*P* = 0.088), although this negative impact is no longer significant after correcting for multiple hypothesis testing. Emails to teachers that contained messages of encouragement also failed to increase student participation, with a −0.038 (SE 0.046) impact on study time. The impacts of all three teacher-level interventions on student knowledge scores are small and not statistically significant, with magnitudes of 0.053 SD or less.

**Fig. 1.**
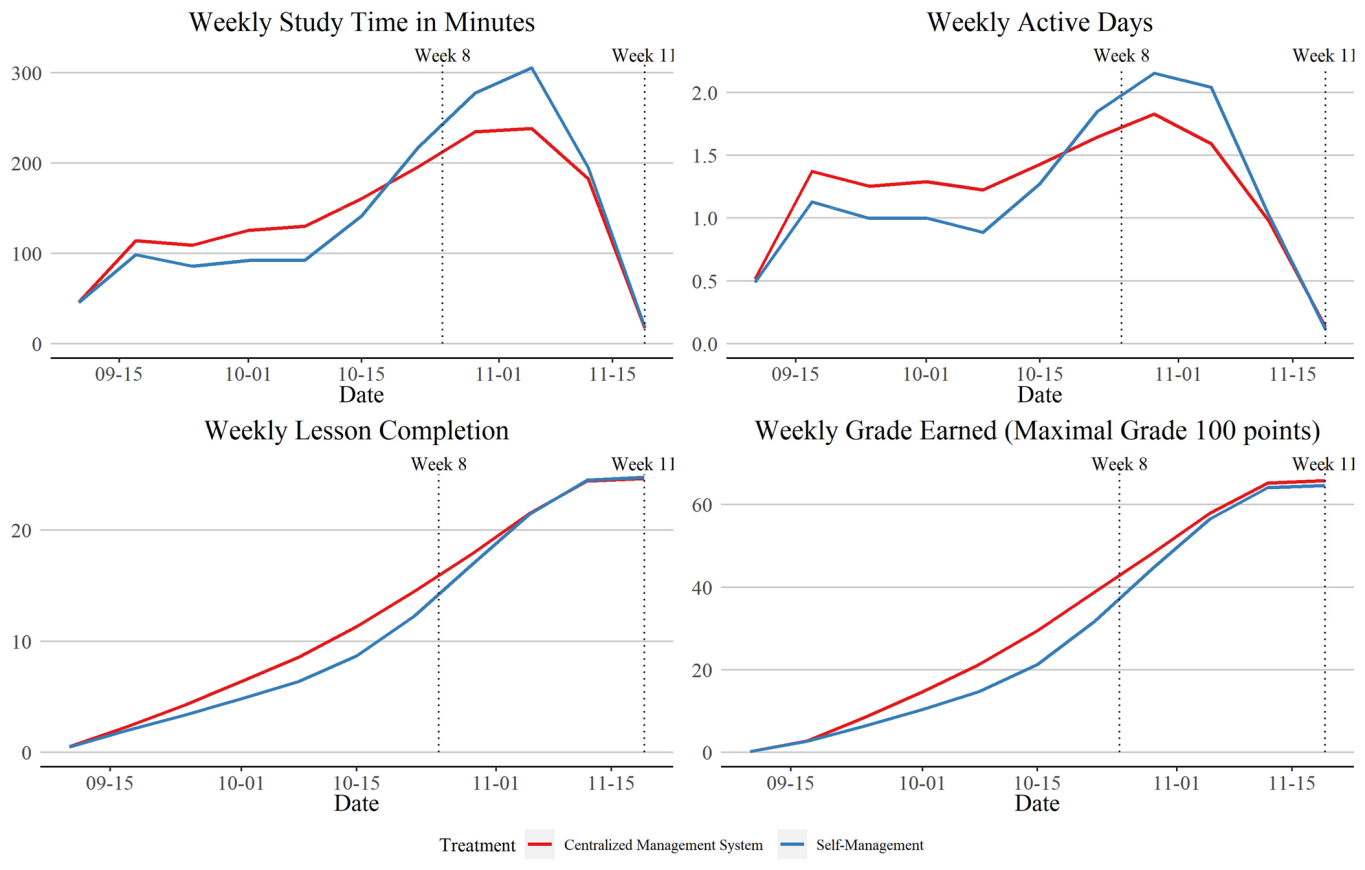
Students in the central management treatment progressed more quickly and evenly through lessons compared to those in self-managed schools. Note: weekly learning dynamics show mean study time on the online platform per week, number of active days on the online platform each week, cumulative number of lessons completed by week, and cumulative grades earned for completing activities on the platform by week.

The strongest impacts of any of our interventions come from our system-level intervention of using centralized management rather than decentralized self-management. After 8 wk of the program, students at schools assigned to centralized management had a 0.21 SD (SE 0.11) increase in study time on the platform. This corresponds to an average of 125 min (SE 69) more time spent on the platform, and 2.1 more lessons completed (SE 1.1) (*SI Appendix*, Table S1). The Ministry of Education re-exerted its central authority by sending all schools strong messages in a formal letter in week 9 to urge schools to get teachers and students to complete the course. Fig. 1 shows that this occurred as students in self-managed schools were spending a lot more time on the platform in an effort to catch up, and so by the end of week 11, the gap in our overall study time index had closed to an insignificant 0.023 SD (SE 0.087). However, students in treated schools scored higher grades on platform tasks during this time (*SI Appendix*, Table S4 and Fig. 1), and then score significantly better on a knowledge test in both week 8 and week 11, with a week 11 impact of 0.126 SD (SE 0.056). This improvement in knowledge occurs for four out of five subjects taught online, the exception being Spanish language (*SI Appendix*, Fig. S1).

We examine further whether centralized compared to decentralized self-management varies in its effect according to characteristics of the students or school. *SI Appendix*, Table S2 shows no significant heterogeneity by student gender or socioeconomic status. Using the machine-learning Policy Tree approach of ref. 21, we do find significant heterogeneity with respect to school characteristics. The algorithm selects a decision rule based on the average test scores in the school in the end of school national examinations, choosing a split that is close to the average test score nationwide. Schools with high test scores perform just as well with decentralized self-management as with centralized monitoring, while those with below-average test scores do notably worse with self-management.

## Discussion

The COVID-19 pandemic caused school systems around the world to rapidly pivot to online education, without a lot of evidence on interventions that could be implemented at scale to encourage student engagement and learning. Each of the interventions tried in our study had theoretical reasons, and some empirical evidence, for why they might work. However, as we have seen, many of these light-touch interventions did not have the intended effects. We discuss several potential reasons for this, before then turning to placing the effect sizes of our more successful interventions in context, and discussing cost-effectiveness.

A first possible reason why impacts of some of these interventions were lower than anticipated was that student engagement with this online content was considerably higher than we feared it might be. The experience of high drop-out with MOOCs was not a good baseline of what to expect with high school students given online courses as part of their assigned coursework. An additional factor behind this may have been that the pandemic reduced the opportunity cost of participating in schooling, since other opportunities for leisure and work were more limited. The consequence was that there was less room for improvement, since many students were already spending a lot of time on the platform and completing many modules even under control conditions.

Second, our results are also consistent with the literature on some of the constraints to scaling promising interventions. Nudge interventions have had weaker impacts when implemented on larger samples, and with less in-person contact (22). Our encouragement messages to students and teachers fall within this category. Likewise, the limited impacts of our planning and household team-up intervention for students is consistent with more recent evidence (5) that plan-making interventions have had more limited impacts at scale than in earlier pilots. Our weak overall effect of the benchmarking intervention for teachers is consistent with the idea that when benchmarking is delivered at scale, gains from those who are performing below average may be offset by negative impacts on initial high-performers (16).

Third, all three teacher interventions encouraged teachers to just get their students through to completion of the course, without emphasizing the importance of students spending sufficient time on each module, nor learning goals. This may have led teachers to tell students to just quickly finish a particular module, even if they had not fully finished the ones before, possibly leading to less time spent studying.

Our two most successful interventions were using lotteries to incentivize students, and the system-level intervention of using centralized management. The student-level lotteries were successful in getting students to spend more time studying, but, even when we also incentivized scores on platform tasks, this was insufficient to lead to significant knowledge gains. At a cost of $0.17 per student, the lotteries are a low-cost way of increasing time spent on the platform. However, if this increased time spent on the platform does not yield higher knowledge for students, then this is not a cost-effective approach for increasing learning, especially if one values the opportunity cost of students’ time.

In contrast, the online centralized management approach appears to be a highly cost-effective way of increasing both time spent on the platform and knowledge. It improves learning outcomes by 0.13 SD at a cost of lower than 60 cents per student. To place this effect size magnitude in context, 0.1 SD is the median effect size on learning outcomes of large educational interventions in low- and middle-income countries, and is the equivalent of how much a student might learn in 71 percent of a year of business-as-usual schooling in grade 12 (23). Or as another comparison point, during the COVID-19 pandemic, a combination of phone calls and SMS messages to parents to support their child’s learning in Botswana cost $19 per child for a 0.12 SD increase in learning (24). Centralized management therefore seems highly cost-effective. Our heterogeneity results are then consistent with the idea that autonomy may have more negative impacts on student performance in low-income settings (19)—while Ecuador is a middle-income country, its underperforming schools with low test scores are more similar to low-income school settings.

Additional suggestive evidence of the importance of system-level interventions comes from comparing the completion rates across our three waves of the experiment. In our first wave, students were asked to complete the activities from home when the pandemic hit, and only 55 percent completed all lessons. In contrast, in the expansion in wave 2, the government required completion of the program in order for students to graduate, and this central mandate resulted in 82 percent of the control group completing all modules. Then in wave 3, graduation was no longer tied to completion, and completion rates in control schools were lower again, at 70 percent.

Our system-level intervention provides almost real-time data on student effort and performance. It took place in a context in which personnel from the Ministry of Education had both the willingness to use these data for monitoring, and tools they could use to hold teachers accountable. For example, they reminded teachers that this course was considered part of their required activities for which they are paid their salary. This is consistent with management reforms improving outcomes in systems in which there is a credible threat of sanctions (25), whereas interventions that have provided information without having incentives or accountability in place have been less successful (26).

Although most schools have now returned to in-person classes for the majority of their teaching, online classes are likely to continue to be part of educational systems going forward. This can include both their use as part of regular programming to enable students to study subjects not offered by their schools and to supplement their regular education, as well as being a toolkit that school systems can rapidly roll-out in times of future health shocks, disasters, and war [e.g., online schooling is currently being used in Ukraine, (27)]. The lessons from these efforts to provide online education in high schools during the pandemic show how schools and students can be incentivized to engage in online learning, as well as highlighting types of low-touch interventions that may not have that much impact.

## Materials and Methods

### Context.

These interventions took place as part of a larger online education project called “Showing Life Opportunities” that was conducted in collaboration with the Ministry of Education in Ecuador. The broader effort aims to evaluate the effectiveness of online courses in entrepreneurial education and science on life outcomes of students. This paper focuses on interventions intended to improve the implementation, by ensuring students took part in the online courses. This project received human subjects approved from the Universidad San Francisco de Quito, Institutional review board (IRB) approval number 2018-208E and by the IRB Committee of Innovations for Poverty Action IRB-USA, IPA IRB Protocol #: 15629 with a waiver of consent.

### Waves and Randomization.

Our interventions took place during three waves. The first wave worked with 108 schools in educational Zone 2 and had teachers supervise the students taking the online material through computer laboratories in their schools. Random assignment of the teacher benchmarking intervention occurred at the school level, with half the schools assigned to treatment. This wave started in September 2019, and finished in July 2020, with the COVID-19 pandemic necessitating a shift to students accessing the material from home after March 2020. The second wave occurred as a rapid response to the pandemic, with the Ministry of Education quickly offering the content to students in 416 schools in the Highlands area of the country, between May 2020 and July 2020 as they finished their interrupted school year. The student-level lottery, plan and team-up within household, and encouragement interventions were randomized at the student level. The teacher administrative SMS and encouragement messages were randomized at the class level, among 532 virtual classes. The third and final wave occurred in the Coastal region of the country, which has a different school calendar, and took place during their new school year from September to November 2020. The system-level centralized school management, and student-level lottery and teaming-up interventions were randomized at the school level, among 598 schools. *SI Appendix*, Fig. S2 illustrates the experimental design and sample sizes in each wave.

### Data and Estimation.

Student and class-level baseline variables were collected via an online survey on the platform prior to any of the interventions beginning. *SI Appendix*, Tables S5–S7 provide descriptive statistics and show baseline means are balanced by treatment status. Our main two outcomes of study time and knowledge are index measures, prespecified in a pre-analysis plan (AEARCTR-0003553 and AEARCTR-0005982) and defined in the *SI Appendix*. Our outcome data on study time consist of administrative data from the platform on the amount of time students spend on the platform and the activities completed. Outcome data on knowledge come from subject knowledge tests given by schools to the students, administered through the online platform. We have 80% of students complete these tests in wave 1, 95% in wave 2, and 88% in wave 3, with attrition uncorrelated with treatment status (*SI Appendix*, Table S8).

We estimate intention-to-treat effects of being assigned to a particular treatment using a regression of the outcome on dummies for treatment assignment, with controls for randomization strata, for the preintervention level of the outcome of interest, and with additional baseline controls using the postdouble selection lasso method of ref. 28. SEs are clustered at the level of the randomization (student, class or school). Heterogeneous effects of the treatments with gender and wealth, and of the benchmarking treatment with initial performance are performed by including the variable and its interaction with treatment in these regressions. We use the policy tree machine learning approach of ref. 21 to examine heterogeneity of the centralized management intervention with respect to school-level characteristics.

Anonymized data and replication code for the analysis in this paper are deposited in the Open Science Framework data repository https://osf.io/kmgvu/ (29).

## Supplementary Material

Appendix 01 (PDF)

## Data Availability

Anonymized survey data data have been deposited in OSF (https://osf.io/kmgvu/) (29). All study data are included in the article and/or *SI Appendix*.

## Footnotes

^*^Television- and radio-based instruction was also used in many countries at the primary and sometimes lower secondary levels, but internet-based digital instruction was the most common policy used in upper secondary (1).
