# Supplementary material for: System-, teacher-, and student-level interventions for improving participation in online learning at scale in high schools
Source: Proc Natl Acad Sci U S A. 2023 Jul 17;120(30):e2216686120. doi: 10.1073/pnas.2216686120 (PMC10372661; doi:10.1073/pnas.2216686120)
Supplement: Supplementary file 1 — Appendix 01 (PDF) [file pnas.2216686120.sapp.pdf]

PNAS

Supporting Information for

System-, Teacher-, and Student-level Interventions for Improving Participation in Online Learning at Scale in High Schools

Igor Asanov, Anastasiya-Mariya Asanov (Noha), Thomas Åstebro, Guido Buenstorf ,
Bruno Crépon, David McKenzie, Francisco Pablo Flores T., Mona Mensmann, Mathis Schulte

Paste corresponding author name here
Email: dmckenzie@worldbank.org

This PDF file includes:

Supporting text
Tables S1 to S11
Figures S1 to S9
SI References

Supporting Information Text

A: Detailed description of the three experimental waves and associated interventions

Context: The public schooling system in Ecuador consists of 6 grades of primary education, three grades of lower secondary (grades 7 through 9), and three grades of upper secondary or high school (grades 10 through 12). Schooling is free, and 75 percent of students attend public institutions. High schools are divided into two specializations: technical and science. In both types of high schools, students have a common core curriculum, and sit a national standardized exam called *Ser Bachiller* to graduate, but they are offered different elective subjects. We work with the technical specialization schools, which offer skills designed for students to be able to directly enter the labor market, including entrepreneurship as an elective subject. In our first wave we also worked with science specialization schools, that focus more on preparation to enter higher education. Our broader project, *Showing Life Opportunities*, is intended to provide personal initiative and negotiations skills to students considering entrepreneurship, as well as offer insights into alternative career pathways through online training in statistics and the scientific method, with English and Spanish online content offered as comparison courses.

Due to climatic reasons, the education system splits the country into two separate regimes which operate on different school calendars. The Highlands-Amazon regime holds classes from September to June, whereas the Coastal regime holds classes from April through to February.

Wave 1: In wave 1 we offered the courses to students in Educational Zone 2, part of the Highlands-Amazon regime. Schools had to have a steady internet connection and enough computers for at least one class to use at a time. We worked in total with 108 schools, containing 560 classes, and 15443 students. The program started on September 23, 2019. It was interrupted by national protests in the country for a few weeks soon after starting, and then resumed until the pandemic closed schools on March 13, 2020. This resulted in a break for a month and a half, before schools then encouraged students to access the materials from their homes and finish the program. 12,232 students reached the endline survey in July 2020 (more than 80% of those registered on the platform).

Wave 1 was used for our teacher benchmarking intervention. Our goal was to have teachers actively support the program by being present in class to oversee their students using the online materials and to encourage usage. We formed matched quadruplets of schools based on the number of classes in the school, number of lessons completed on the platform to date, whether the students were actively using the platform, and the subjects being provided to their students. Within each quadruplet, we randomly allocated two schools to the benchmarking treatment and two to control (see Figure S2). Table S5 shows balance on baseline school and student characteristics for the two groups.

The benchmarking intervention then took place over 6 months between December 2019 and May 2020. Each treated teacher received 20 personalized benchmarking emails on their verified email that they used to access the online educational platform. These emails showed the number of lessons completed, number of unfinished lessons, and last active

lesson for the teacher's class(es) and compared it to those of other classes doing the same type of course. This information was presented graphically, with the help of bar plots. Figure S3 provides an example.

Wave 2: The COVID-19 pandemic closed schools on March 13, 2020. The Ministry of Education of Ecuador then quickly looked for content it could provide to help its students in the Highlands/Amazonia Regime complete their school year, which was to finish at the end of June. We worked with them to offer the courses that had been offered in wave 1 to the remaining seven educational zones in the Highlands/Amazonia. This was offered to students in the final year (grade 12), and was made a requirement for graduation for these students. The courses were offered in 416 schools and 14,398 students started the program, with 13,714 students (more than 95%) reaching the endline survey by the end of July 2020.

This program was offered on a compressed schedule, with students starting May 18, 2020, and needing to finish by the end of June 2020. We implemented student- and teacher-level interventions to try to ensure students were able to complete the course on time.

The student-level interventions started June 1, 2020, and were focused on students who were not already rapidly advancing through the material. Students who had completed more than 6 lessons in the first two weeks it was offered were screened out, and then the remaining 11834 students who were enrolled in the online platform, but not progressing as fast, were randomized at the individual level into one of four groups (Figure S2):

(1) a control group of 1961 students, who would continue to use the platform at their own pace.
(2) a lottery intervention group of 3936 students. Students were told they would win a lottery ticket each time they finished a lesson. Figure S4 provides an example. Given the emergency context and short period to complete the course, these incentivized completion only, and not performance on other outcomes such as knowledge tests. The winners of the lottery received $20.
(3) an encouragement messages group of 3975 students. Students were shown encouragement messages on the platform, selected at random from a set of messages designed to convince students that they could still finish the course despite the hurdles of the pandemic. These were intended to change beliefs about the feasibility of learning, and to help students keep negative effects in perspective. Figure S4 provides examples of messages such as #DearClassOf2020, "Graduating is a great achievement. Thank you for your drive to shape the world into something better. Source: UNICEF. You can finish all the lessons" or "Pandemic is temporary, what you do will always last."
(4) a plan and within-household team-up group of 1962 students. Students were asked to make plans for how they will study and share these plans with others in their house. Figure S4 gives an example of the instructions. Students were asked to share their notes on number of hours spent already, reflect on what they had learned, and plan their next study sessions, as a way of increasing the chance of students using self-regulated learning strategies.

Table S6 shows baseline characteristics and balance for the student-level interventions.

The teacher-level interventions were introduced on June 17, 2020, one month after students had started the program, and were intended to spur teachers to help stimulate their students to complete the program and graduate on time. Randomization occurred at the teacher level, with 532 teachers (classes) in 416 schools formed into strata based on level of activity, educational zone, number of students in the platform, type of course students were assigned to, and number of lessons completed by students to date. Figure S2 shows how teachers (and their respective students) were randomly assigned into three groups:

(1) A control group of 178 teachers and 5031 students, where no messages were delivered to teachers.

(2) An administrative SMS group of 177 teachers and 4693 students. These administrative messages acted as a combination of reminders and increases in the salience of monitoring. The message sent read "*Remember, your Zone [Name] delegate and MINEDUC monitor your activity in the SLO project. Make sure your students finish until the end of the school year. SLO*". (SLO is an acronym for our online program "Showing Life Opportunities"). Monitoring was common knowledge as ministry personnel were shown on the class dashboard of the online platform next to each teacher in each class. We hoped that the administrative SMS would increase the salience of this monitoring, as well as reminding the teacher of the need to finish on time.

(3) An encouragement email group of 177 teachers and 4674 students. Teachers in this group were sent emails encouraging them to watch a video[1] of the successful completion of the program by students in Zone 2, and telling them that we hoped that their students would finish the program successfully in time for the end of the school year. Figure S5 provides an example.

Table S5 shows balance and baseline characteristics for these teacher-level interventions.

Wave 3: The final wave of the experiment took place in schools in the 9 educational zones in the Coastal Regime. The new school year for these students started later than usual in June 2020, and the government had more time to prepare for launching online schooling for these students without the pressure of the end of the school year facing the Highlands-Amazonia students. Our study then provides evidence of incentives to learn under a "new normal" of remote studying and learning from home, after the initial shock of the pandemic had lessened. We offered the program to 16,547 students in 598 schools, starting September 7, 2020 and ending November 16, 2020. 14,562 students (more than 88% of those registered) completed the endline survey on the platform. Building on our previous experiments and lessons, we tested more interventions at the student level, as well as one at the system level.

[1] We sent encouragement emails with a link to a YouTube video of the successful completion of the program to 177 treated teachers. We hit 276 views of this video during the time of the intervention in Ecuador. We observe spikes in views of this video each time encouragement emails were sent. On average this video was watched for 1 minute 12 seconds (~44 percent of total time of the video: 2 minutes 43 seconds).

We formed strata of schools based on type of course offered, educational zone, cluster size (number of students enrolled), student average performance on the state exam at the end of school (Ser Bachiller) and the number of students in the 12th grade. We then randomized schools into the following student-level and system-level interventions:

(1) Centralized monitoring through an online learning management system. 149 schools containing 3882 students were placed in this treatment group. Here Ministry of Education administrative personnel (heads of zones, the central office) would have access to the online management system and could see real-time information about take-up and usage of the educational platform in each school, and would receive weekly reports. They could then use these to monitor teachers in poor performing schools and encourage them to improve. Figure S6 provides an example of the system and weekly report.

(2) Self-management: 150 schools containing 4125 students were instead assigned to self-manage their participation in the program and the progress of these students. Ministry of Education officials were not provided the centralized information and weekly reports on these schools, and it was up to each individual school and teacher within school to ensure that their students progressed. Teachers receive the same information in this condition as in centralized management, the difference is in whether the central ministry also receives it.

(3) Student lotteries for lesson completion and score. The early results from wave 2 had suggested that students were responding to the lottery incentives, but there was a concern that they might be rushing to finish lessons without caring as much what they learned. For wave 3, 149 schools containing 4025 students were assigned to a new lottery program. Here students earned a ticket for a lottery prize for each completed module, and also tickets for their performance on platform tasks. Students were sent emails (Figure S7) to inform them of this, and a weekly draw was held, with one winner per school receiving $10. Note that these schools were also subject to the Centralized monitoring as in group 1, and so we measure the additional effect of student lotteries on top of centralized monitoring.

(4) Encouragement to team-up with peers remotely. In wave 2, there was insufficient time to get students to pair up with others in their class, which is why they were asked to share plans with household members. In wave 3, we wanted to see whether working with classmates remotely would create peer incentives to complete the program. 4515 students in 150 schools were assigned to get emails (Figure S8) encouraging them to work with peers to complete as many lessons as possible and get a better score. Again these schools were also subject to Centralized monitoring, so that we measure the additional impact of peer team-ups.

Table S8 shows baseline characteristics of these four groups in the Coastal Regime and balance across treatments.

Our three waves of the experiment cover schools in different parts of the country. Table S9 compares the baseline characteristics of these schools, and their teachers and students across the three waves. Although there are some differences in means, there is considerable overlap in the distributions, and lessons are all taking place within the same school system in the same country. As a check that differences across waves are due to differences in the treatments, and not to differences in the characteristics of the schools, teachers, and

students, we use these characteristics in Table S9 to reweight the data using inverse propensity scores in order to make all waves look like wave 3, the Coastal Region. Table S10 shows that this results in only minor changes in the magnitude of the estimated coefficients, and does not change any of our substantive conclusions.

B: Definitions and Measurement of Main Outcomes

We construct **study time index** as a z-score based on the following variables: study time on the platform (in minutes), active days on the platform, and number of lessons completed. These variables are captured instantly and automatically on the online platform (based on any input-response by user). We construct **knowledge index** as a z-score based on the performance on five subject-specific knowledge tests executed via the online platform: Statistics and Scientific Thinking, Negotiations, Personal Initiative, English, Spanish. Students' correct answers coded as one in each item of each knowledge test. Both indexes are an average z-score over standardized z-score of variables in the respective family of outcomes described above (z-scores are standardized with respect to the control group mean). These indexes are constructed for standardization purposes and to account for multiple hypothesis testing within family of outcomes.

In addition to these main outcomes, we use a pre-specified set of baseline (pre-intervention variables) as potential control variables that we then select from using the post-double selection Lasso method of (1). This list consists of:

- *Two class-level variables:* the total number of active students on the platform who have started at least one lesson; and whether the class has at least three students in it who have started at least one lesson (active class). These variables are not used in the Coastal Regime regressions as controls, since random assignment took place before students had started on the platform.
- *A wide range of individual-level variables:* Approximately 70 individual-level characteristics of students were used as potential controls (with small differences across the three waves depending on measurement). These include demographic variables (gender, age); socioeconomic status and family background (e.g. main language spoken, parental durable assets owned, parental education and occupation status); attitudes, knowledge, and intentions towards careers in entrepreneurship (e.g. interest in entrepreneurship, perception of it as a career, study plans in this field, knowledge of others in this field); attitudes, knowledge, and intentions towards careers in STEM (e.g. interest in STEM, perception of STEM careers, study plans in this field, knowledge of others in this field); preferences and psychological profile (e,g. personal initiative, risk preferences, Big-5 personality traits; trust; creativity, cognitive reflection, grit); and baseline subject knowledge

C: Empirical Approach

We estimate the impact of being assigned to treatment T on outcome Y for student i in school or class j through the following pre-specified Ancova regression:

$$Y_{i,j,1} = \beta_0 + \beta_T T_{i,j} + \mu Y_{i,j,0} + \lambda' controls_{i,j} + \theta' strata_{i,j} + \varepsilon_{i,j} \qquad (1)$$

Where $Y_{i,j,0}$ is the baseline (pre-intervention) measure of the outcome variable (e.g. baseline knowledge index, or amount of time spent studying on the platform prior to interventions being launched); *controls* are a vector of control variables selected using the post-double selection lasso method of (1); *strata* are a vector of dummies for the different randomization strata; and the error term $\varepsilon_{i,j}$ is then clustered at the level of random assignment. When there are multiple treatments randomized within the same level (student, teacher or system) and experimental wave, we include dummies for assignment to each treatment in equation (1). For example, we have three student-level interventions in wave 2, and so include dummies for assignment to the lottery treatment, assignment to the plan and team up within household treatment, and for assignment to the encouragement messages treatment in this equation.

The post-double selection lasso method to choose controls serves two purposes. First, it offers the potential to boost statistical power by choosing covariates that are correlated with the end outcomes of interest. Second, to the extent that there are chance imbalances from the randomized assignment, or imbalances resulting from selective attrition, it can correct bias. In practice, we find attrition is generally uncorrelated with treatment status (Table S8), our sample sizes are large and balanced on observables (Tables S5-S7), and once we have controlled for the baseline outcome and randomization strata, the remaining variables do not have strong predictive power. The result is that our estimates with these controls are similar in magnitude and precision to those that would be obtained without using this method, and few covariates are typically selected for inclusion.

Our main approach to multiple hypothesis testing is to restrict the number of outcomes of interest by focusing on two index measures: study time, and knowledge, defined above. Then, since our interventions occur at different levels (system, teacher, and student), and in different settings and experimental waves, we view each intervention as a separate policy choice of interest. We then follow the recommendation of (2) in not adjusting for multiple treatments across experiments. This allows our planning treatment to be compared to the results of other planning treatments in the literature, our lottery treatments to be compared to the results of other experiments offering students financial incentives, etc. In addition, we calculate sharpened q-values to control the FDR to account for our two main outcomes (study time and knowledge test indexes) and multiple interventions within each different combination of waves and levels of intervention (see Figure S2).

We estimate heterogeneity of treatment effects using two approaches. First, following the suggestion of previous literature (e.g. (3)), we hypothesized and pre-registered the hypothesis that impacts of benchmarking may vary depending on whether teachers were initially above- or below-average. We therefore add a variable for whether the teacher had above average performance and its interaction with treatment to equation (1), and report the results in Table S3. We use the same approach of including a covariate and its interaction with treatment for examining whether impacts vary with student gender and socioeconomic status, although theory offers less clear predictions about what type of heterogeneity one should expect in these cases (Table S2).

The second approach we use to examine heterogeneity in impacts is used for our system-level intervention. The goal is to help understand which groups of students and schools policymakers should use this policy for, and we use the policy tree machine-learning algorithm of (4). We allow the algorithm to consider eight variables from our administrative data (type of school funding, type of teacher contract, school performance level) that the Ministry of Education collects regularly and can thus use for decision-making. We do this for the outcome of knowledge gains in week 11.

This results in a simple decision tree, shown in Figure S9. The algorithm selects for a decision rule based on the average score in the school in the national end of school examininations (*Nota SER Bachiller)*. High-performing schools (those with a grade above 7.68) can be self-managed, but low-performing ones should be subject to centralized management. By way of comparison, the national average grade on this examination in our sample of schools is 7.39. For comparison, we then also re-run the same algorithm for the week 8 outcomes of study time index and performance index. The algorithm again selects a decision rule with a cut based on grade on this exam, albeit with slightly different cutoffs.

D. Measuring cost-effectiveness

To investigate cost-effectiveness we focus on impacts on the knowledge index. We first compute effect size as the mean difference between treatment groups divided by the standard deviation of the control group (SD). We then compute the cost of each intervention in a comprehensive way, including the direct cost of delivering the interventions but also the indirect costs associated with shaping the interventions and running the interventions. This is thus a broad notion of an average cost per final user.

Table S9 below presents the results. For example, the Centralized learning management system compared to Self-management improves knowledge by 0.126 SD with costs per targeted participant of $0.509. This large improvement in learning outcomes taken together with low costs leads to a cost-effectiveness ratio of the centralized learning management system of $4.04 per SD. Another intervention with a large cost-effectiveness is the Encouragement SMS on the Teacher level intervention which reaches a cost effectiveness ratio of 6.37, although we note that this impact is not statistically significant. None of the student level interventions had a statistically significant impact on knowledge. The encouragement message on screen has the lowest cost-effectiveness ratio (5.47) among student level interventions based on the point estimates.

Table S1: Components of increase in study time from successful interventions

	Dependent variable:		
	Time On The Platform	Active Days	Lessons Completed
	(1)	(2)	(3)
**Panel A: Lottery ticket for lesson completion**			
Lottery	76.034	0.094	0.047
	(24.925)***	(0.090)	(0.096)
Control Group Mean	1418.121	8.225	17.9
Students	11834	11834	11834
**Panel B: Lottery ticket for lesson completion and score**			
Lottery	91.207	0.406	0.928
	(42.793)**	(0.287)	(0.362)**
Control Group Mean	1348.401	8.515	17.9
Schools	448	377	377
Students	12422	12320	12320
**Panel C: Centralized monitoring (Week 8)**			
Centralized Monitoring	125.160	1.052	2.064
	(68.749)*	(0.620)*	(1.085)*
Control Group Mean	798.13	7.34	11.86
Schools	598	598	598
Students	16547	16547	16547

Note: We omit control variables in tables for the clarity of presentation, but all regressions include controls selected with a double-lasso procedure. In panel B and C, standard errors are clustered at the school-level (level of randomization in wave 3 experiment).*p<0.1; **p<0.05; ***p<0.01

Table S2: Impacts of student and system interventions do not vary with student gender and socioeconomic status

	Encouragement	Lottery	Team-Up	Lottery	Team-Up	Centralized Management
	(1)	(2)	(3)	(4)	(5)	(6)
Panel A: Heterogeneity of Impact on Study Time with Gender						
Treatment	0.051	0.087***	0.053	0.147**	0.072	0.158
	(0.034)	(0.032)	(0.035)	(0.071)	(0.062)	(0.117)
Female	0.246***	0.246***	0.246***	0.082*	0.082*	0.096*
	(0.046)	(0.046)	(0.046)	(0.045)	(0.045)	(0.042)
Assigned to Treatment*Female	-0.035	-0.010	-0.021	0.010	0.039	0.096
	(0.055)	(0.051)	(0.063)	(0.070)	(0.059)	(0.063)
Panel B: Heterogeneity of Impact on Study Time with SES						
Treatment	0.030	0.054	0.041	0.169***	0.109*	0.231**
	(0.037)	(0.040)	(0.035)	(0.065)	(0.059)	(0.117)
Income Above Median	-0.039	-0.039	-0.039	0.010	0.010	-0.095***
	(0.041)	(0.041)	(0.041)	(0.042)	(0.042)	(0.036)
Assigned to Treatment*Income Above Median	0.012	0.057	0.005	-0.062	-0.067	-0.041
	(0.048)	(0.050)	(0.059)	(0.062)	(0.057)	(0.049)
Control Group Mean	0			0		0
Schools	-			448		598
Students	11834			12442		16547

Table S3: The teacher benchmarking intervention had heterogeneous effects depending on their starting point

	Dependent variable:		
	Lessons Completed	Time On The Platform	**Study Time Index**
	(1)	(2)	(3)
Benchmarking	-0.871	-70.767	-0.128
	(0.527)*	(48.461)	(0.077)*
	p = 0.099	p = 0.145	p = 0.097
Lessons Completed Lower Than Mean	1.528	92.120	0.134
	(0.714)**	(45.622)**	(0.091)
	p = 0.033	p = 0.044	p = 0.139
Benchmarking * Lessons Completed Lower Than Mean	1.635	86.435	0.199
	(0.832)**	(58.378)	(0.109)*
	p = 0.050	p = 0.139	p = 0.068
Control Group Mean	14.595	784.78	0
Schools	106	106	106
Students	15443	15443	15443

Note: We omit control variables in tables for the clarity of presentation, but all regressions include controls selected with a double-lasso procedure. Standard errors are clustered at the school level. The take-up outcomes (Lessons Completed, Time Studying) were recorded during the intervention. *p<0.1; **p<0.05; ***p<0.01

Table S4: Students in Centrally managed schools scored higher on platform tasks

	Dependent variable:		
	Average Grade Week 8	Project Completed Week 8	**Performance Index Week 8**
	(1)	(2)	(3)
Centralized Monitoring	6.516	0.021	0.184
	(3.204)**	(0.019)	(0.100)*
	p = 0.043	p = 0.282	p = 0.067
Control Group Mean	30.77	0.06	0
F-test of Joint Significance	0.1	0.46	0.18
Schools	598	598	598
Students	16547	16547	16547

Note: We omit control variables and non-system level interventions for this wave (Lottery, Team Up) in tables for the clarity of presentation. All regressions include controls selected with a double-lasso procedure. Standard errors are clustered at the school level. The performance outcomes (Average Grade, Project Completed) were recorded during the intervention. *p<0.1; **p<0.05; ***p<0.01

Table S5 - Wave 1: Balance on the outcome variables measured at the baseline

Variable Name	Mean in Control Group	Mean in Benchmarking Group	p-value corrected
**Study Time Outcomes at Baseline**			
Time on the Platform B	288.47	317.22	1
Lessons Completed B	5.64	6.67	0.317
Study Time Index B	-0.09	0.11	0.705
**Knowledge Outcomes at Baseline**			
Knowledge – Statistics B	0.02	-0.02	0.768
Knowledge – Spanish B	0.01	0.02	1
Knowledge – English B	0.03	0.02	1
Knowledge Index B	-0.01	0.01	1
**Attitudes and Intentions at Baseline**			
Entrepreneurial Attitude B	0.01	0.02	1
Entrepreneurial Intentions B	0	0.02	1
STEM Attitude B	0.01	0.01	1
STEM Intentions B	-0.01	0.01	1
Attitudes and Intentions Index B	0.01	-0.01	1
Joint Orthogonality Test (Prob >F)			0.14

Note: Baseline outcome variables were collected before the intervention. Estimation made with standard errors clustered on the school level, controlling for the missing values. Each p-value is obtained by regressing the treatment on the given baseline outcome. p-values are corrected per treatment with the Holm correction.

Table S6 – Wave 2: Balance on the outcome variables measured at the baseline for the Teacher- and Student-Level interventions

	Teacher Level Interventions					Student Level Interventions						
Variable Name	Mean in Control Group	Mean in Email Group	Email p-value corrected	Mean in SMS Group	SMS p-value corrected	Mean in Control Group	Mean in Lottery Group	Lottery p-value corrected	Mean in Encourag. group	Encourag. p-value corrected	Mean in Team Up Group	Team Up p-value corrected
**Study Time Outcomes at Baseline**												
Time on the Platform B	1413.11	1391.78	1	1417.63	1	525.14	532.35	1	521.31	1	533.92	1
Active Days B	10.37	10.22	1	9.94	1	4.64	4.64	1	4.57	1	4.73	1
Study Time Index B	0.02	-0.01	1	-0.01	1	0	0	1	-0.02	1	0.02	1
**Study Speed Outcomes at Baseline**												
Entries B	51.68	51.72	1	53.46	1	23.66	22.09	1	20.75	0.103	23.67	1
Lessons completed per day B	2.03	2.02	1	2.1	1	1.72	1.66	0.920	1.68	1	1.7	1
Study Speed Index B	-0.02	-0.02	1	0.04	0.804	0.04	-0.01	0.576	-0.03	0.124	0.03	1
**Knowledge Outcomes at Baseline**												
Knowledge - Statistics	0.01	-0.02	1	0.02	1	0.02	0	1	-0.01	1	-0.01	1
Knowledge - Spanish	-0.01	0.01	1	0.02	1	0.02	-0.01	1	0	1	0	1
Knowledge Index B	0	-0.01	1	0.02	1	0.02	0	1	-0.01	1	-0.01	1
Joint Orthogonality Test (Prob >F)			0.56		0.24			0.23		0.102		0.925

Note: Baseline outcome variables collected before the intervention. Estimation made with standard errors clustered on teachers' level for teacher level interventions. Note that course personal initiative and negotiations knowledge are not present at this balance table as they were not measured before the course. Each p-value is obtained by regressing the treatment on the given baseline outcome. p-values are corrected per treatment with Holm correction.

Table S7 – Wave 3: Balance on the outcome variables measured at the baseline in System-level and Student-level interventions

Variable Name	Mean in Centralized Monitoring Group	Mean in Self-Management Group	Self_Management p-value corrected	Mean in Lottery Group	Lottery p-value corrected	Mean in Team-Up Group	Team Up p-value corrected
**Study Time Outcomes**							
Time on the Platform B	395.51	329.75	1	374.16	1	417.84	1
Active Days B	4.27	3.48	1	3.86	1	4.47	1
Lessons completed B	6.16	4.64	1	5.52	1	6.35	1
Study Time Index B	0.07	-0.16	1	-0.03	1	0.12	1
**Performance Outcomes**							
Average grade B	14.47	10.14	1	11.83	1	14.19	1
Project completed B	0	0	1	0	1	0	1
Performance Index B	0.1	-0.13	1	-0.04	1	0.08	1
**Knowledge Outcomes**							
Knowledge - Statistics	0.03	0.03	1	0	1	0	1
Knowledge - Spanish	0.02	0.01	1	0.01	1	0.03	1
Knowledge - English	0.04	0.02	1	0.05	1	0.04	1
Knowledge Index B	0.01	-0.01	1	0	1	0	1
Joint Orthogonality Test (Prob >F)			0.53		0.1		0.18

Note: Baseline outcome variables collected before the intervention. Estimation made with standard errors clustered on school level, controlled for the missing values. Note that course personal initiative and negotiations knowledge are not present at this balance table as they were not measured before the course. Each p-value is obtained by regressing the treatment on the given baseline outcome. p-values are corrected per treatment with Holm correction.

Table S7b – Wave 3: Balance on the administrative control variables in System-level and Student-level interventions

Variable Name	Mean in Control Group	Mean in Self-Management Group	Self-Management p-value corrected	Mean in Lottery Group	Lottery p-value corrected	Mean in Team-Up Group	Team Up p-value corrected
Zona 1	0.15	0.11	0.970	0.11	1	0.11	1
Zona 2	0.01	0.01	1	0.01	1	0.01	1
Zona 3	0	0.02	1	0.02	1	0.01	1
Zona 4	0.22	0.21	1	0.2	1	0.2	1
Zona 5	0.28	0.29	1	0.3	1	0.32	1
Zona 6	0.02	0.04	1	0.02	1	0.02	1
Zona 7	0.13	0.19	0.733	0.2	0.392	0.16	1
Zona 8	0.19	0.13	0.695	0.13	1	0.17	1
Zona 9	0	0.01	1	0	1	0	1
Rural location	0.36	0.36	1	0.32	1	0.29	1
Urban location	0.64	0.64	1	0.68	1	0.71	1
Type of School Funding (Fiscal)	0.91	0.93	1	0.95	1	0.92	1
Number of students in school	243.4	249.05	1	242.91	1	264.81	1
Number of students K12 in Technical Special.	73.28	74.11	1	72.15	1	79.7	1
Average performance at state exam (ser_bachiller)	7.26	7.29	1	7.25	1	7.4	1
Number of entrepreneurship teachers	1.61	1.55	1	1.54	1	1.69	1
Teacher's email not verified by the program	0.04	0.04	1	0.05	1	0.01	1
Teachers with fixed contracts	0.78	0.78	1	0.7	1	0.77	1
Teachers with temporary contracts	0.21	0.22	1	0.3	1	0.23	1
Joint Orthogonality Test (Prob >F)			0.68		0.29		0.85

Note: Balance on school level calculated with administrative data on schools.

Table S8: Attrition in knowledge outcome is uncorrelated with treatment status

Participation	Share of Students who Participated in Knowledge Test	p-value (difference from control)
Panel A: Experiment 1 (Zone 2)		
Control (Teacher Level)	0.790	
Benchmarking (Teacher Level)	0.804	0.50
Panel B: Experiment 2 (Highland Regime)		
Control (Teacher Level)	0.956	
Encouraging Email(Teacher Level)	0.957	0.84
Monitoring SMS (Teacher Level)	0.944	0.65
Control (Student Level)	0.984	
Lottery (Student Level)	0.986	0.74
Encouragement (Student Level)	0.987	0.59
Team Up with Household Member (Student Level)	0.992	0.02
Panel C: Experiment 3 (Coastal Regime)		
Central Management week 8 (System Level)	0.501	
Self-Management week 8 (System Level)	0.461	0.46
Lottery week 8 (Student Level)	0.498	0.81
Team Up with a Peer week 8 (Student Level)	0.547	0.37
Central Management week 11 (System Level)	0.869	
Self-Management week 11 (System Level)	0.876	0.94
Lottery week 11 (Student Level)	0.877	0.67
Team Up with a Peer week 11 (Student Level)	0.896	0.11

Note: Attrition in the treatment groups in experiment 1 (zone 2), experiment 2 (highland regime) and experiment 3 (coast regime). The estimations in experiment 1 and experiment 3 are made with standard errors clustered at the school level and the estimations in experiment 2 (teacher level) are made with standard errors at the school level. We observe slight attrition issue in the plan and team-up treatment, which is interesting given the result of (5) who finds that peer mentoring increased registrations. However, given that we do not see any changes in outcomes due to the plan and team treatment (see results section), it is unlikely that is of concern.

Table S9: School-,Teacher-, Student-Level Descriptive Statistics per Experimental Wave

	N	Mean	Std. Dev.	10th	25th	Median	75th	90th
**Wave 1**								
School-Level								
Rural	102	0.2	0.4	0	0	0	0	1
Mean Class Size per School	106	28.7	9.5	16.5	23	29	34	38
State Exam Score	99	7.7	0.6	7.1	7.3	7.7	8.1	8.4
Teacher-Level								
Entrepreneurship Teachers	106	1.3	0.8	1	1	1	1	2
Mean Class Size per Teacher	168	27.7	10.5	14.8	21.9	28	34.1	39.8
Student-Level								
Female	15594	0.5	0.5	0	0	1	1	1
Age	15594	16	1.2	15	15	16	17	18
University Intention	15487	0.7	0.5	0	0	1	1	1
Income above Median	15345	0.6	0.5	0	0	1	1	1
Statistics Score	14722	29.6	26.2	0	0	33.3	33.3	66.7
Spanish Score	14702	52.7	14.6	30.8	46.2	53.8	61.5	69.2
English Score	14644	44.7	24.4	16.7	33.3	50	66.7	83.3
**Wave 2**								
School-Level								
Rural	334	0.4	0.5	0	0	0	1	1
Mean Class Size per School	334	34.7	39.4	4	10	20.5	47	78.7
State Exam Score	334	7.4	0.8	7	7.2	7.5	7.7	8
Teacher-Level								
Entrepreneurship Teachers	217	1.8	1.1	1	1	1	2	3
Mean Class Size per Teacher	532	27.1	38	1	2	15	36	68
Student-Level								
Female	14287	0.4	0.5	0	0	0	1	1
Age	14287	18.1	3.4	17	17	17	18	19
University Intention	14268	0.7	0.5	0	0	1	1	1
Income above Median	14014	0.5	0.5	0	0	0	1	1
Statistics Score	14133	36.9	27.7	0	16.7	33.3	66.7	66.7
Spanish Score	14130	57.7	12.6	38.5	46.2	61.5	69.2	72.7
English Score	14117	53	24.1	16.7	33.3	50	66.7	83.3
**Wave 3**								
School-Level								
Rural	491	0.3	0.5	0	0	0	1	1
Mean Class Size per School	491	27.3	31.2	3	8	17	34	64
State Exam Score	491	7.3	0.9	6.9	7.1	7.3	7.6	8
Teacher-Level								
Entrepreneurship Teachers	491	1.7	1.2	1	1	1	2	3
Mean Class Size per Teacher	622	26.4	30.3	3	8	16	33	61
Student-Level								
Female	16440	0.5	0.5	0	0	0	1	1
Age	16440	17.3	2.4	16	17	17	17	18
University Intention	16404	0.8	0.5	0	1	1	1	1
Income above Median	16201	0.5	0.5	0	0	0	1	1
Statistics Score	16073	35.1	27.1	0	0	33.3	66.7	66.7
Spanish Score	16055	58.8	13	38.5	53.8	61.5	69.2	76.9
English Score	16008	48.3	23.8	16.7	33.3	50	66.7	83.3

Table S10: Robustness of Main Results to Reweighting to Increase Comparability Across Waves

	Original		Reweighted	
	Study Time Index	Knowledge Index	Study Time Index	Knowledge Index
**Student-Level Interventions**				
Lottery ticket for lesson completion	0.083***	0.007	0.068*	0.007
	(0.028)	(0.024)	(0.035)	(0.030)
p-value	0.003	0.753	0.053	0.807
Encouragement messages on screen	0.036	0.025	0.011	0.045
	(0.028)	(0.024)	(0.035)	(0.029)
p-value	0.204	0.282	0.755	0.127
Plan and within household team-up	0.043	0.008	0.037	-0.004
	(0.031)	(0.026)	(0.040)	(0.036)
p-value	0.159	0.771	0.356	0.92
Lottery ticket for lesson completion and score	0.107**	-0.064*	0.107**	-0.064*
	(0.053)	(0.039)	(0.053)	(0.039)
p-value	0.045	0.100	0.045	0.100
Team-up with a peer	0.072	-0.040	0.072	-0.040
	(0.047)	(0.036)	(0.047)	(0.036)
p-value	0.120	0.271	0.120	0.271
**Teacher-Level Interventions**				
Benchmarking emails	-0.023	0.025	0.001	0.046
	(0.071)	(0.033)	(0.068)	(0.036)
p-value	0.745	0.435	0.992	0.206
Administrative SMS	-0.076*	-0.016	-0.045	-0.047
	(0.044)	(0.042)	(0.045)	(0.040)
p-value	0.088	0.71	0.327	0.236
Encouragement emails.	-0.038	0.053	-0.013	0.04
	(0.046)	(0.039)	(0.043)	(0.038)
p-value	0.405	0.178	0.763	0.291
**System-Level Intervention**				
Centralized Monitoring: week 8	0.211*	0.108**	0.211*	0.108**
	(0.114)	(0.042)	(0.114)	(0.042)
p-value	0.065	0.011	0.065	0.011
Centralized Monitoring: week 11	0.023	0.126**	0.023	0.126**
	(0.087)	(0.056)	(0.087)	(0.056)
p-value	0.792	0.025	0.792	0.025

Note: Original estimates are as per Table 1. Reweighted estimates use inverse propensity-scores (within common support) to weight data so that characteristics of schools, teachers, and students in waves 1 and 2 are similar to those in wave 3. We exclude participants with propensity scores below 0.05 or above 0.95, as recommended by (29).

Table S11: Cost, Effect size and cost effectiveness

	Wave	Cost	Effect size	Cost-Effectiveness
**Student-Level Interventions**				
Lottery ticket for lesson completion	2	0.169	0.007	24.109
Encouragement messages on screen	2	0.137	0.025	5.467
Plan and within household team-up	2	0.277	0.008	34.614
Lottery ticket for lesson completion and score	3	1.525	-0.064	-23.828
Team-up with a peer	3	0.433	-0.040	-10.825
**Teacher-Level Interventions**				
Benchmarking emails	1	0.305	0.025	12.215
Administrative SMS	2	0.224	-0.016	-14.017
Encouragement emails.	2	0.337	0.053	6.367
**System-Level Intervention**				
Centralized Monitoring: week 11	3	0.509	0.126	4.040

Cost: Average cost per user in dollars. Broad notion of cost including shaping the intervention, delivering it and the costs of running the interventions.
Effect size: Treatment Control difference divided by the standard error of the knowledge index (regression based coefficients).
Cost effectiveness: Ratio of the cost per user to the standardized effect

Figure S1: Impact of Centralized Management on Subject Test Scores

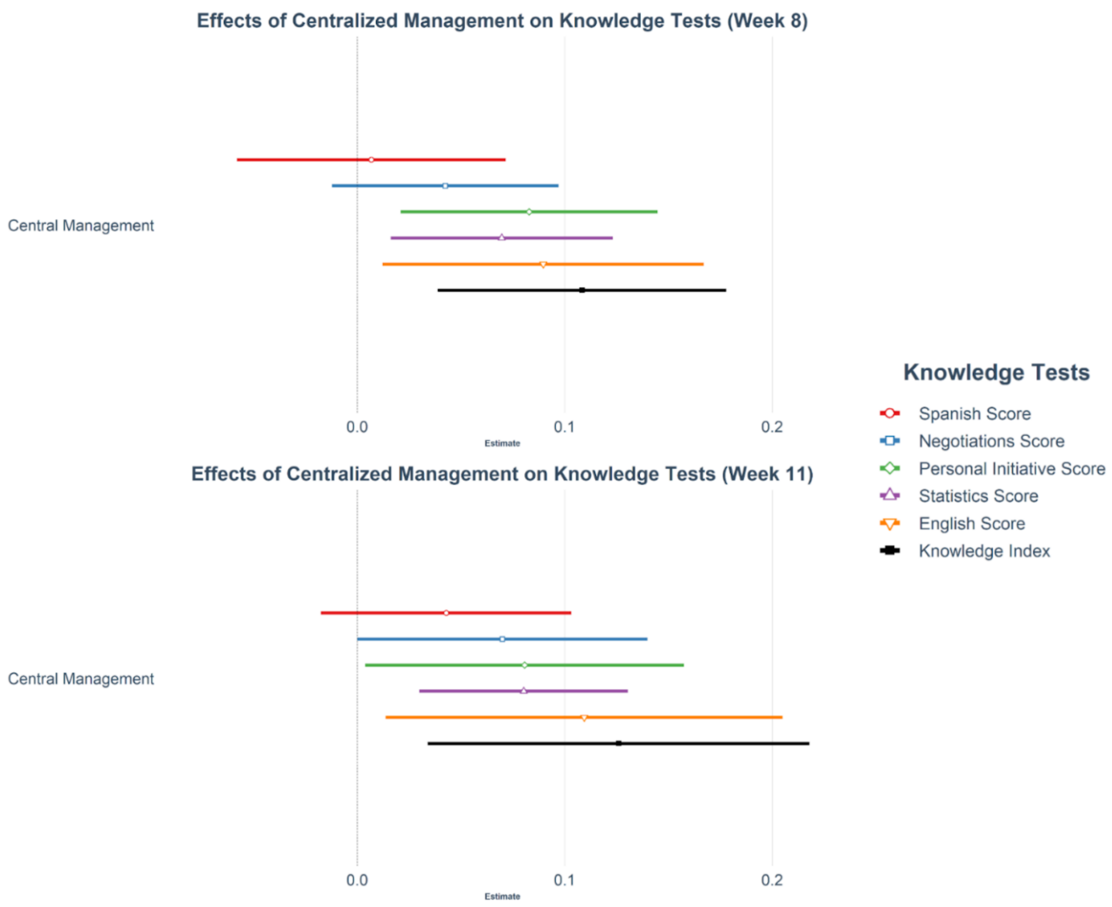

Figure S2: Experimental Design and Sample Sizes

Experimental Wave	Wave 1		Wave 2							Wave 3			
	Zone 2 Experiment		Highlands Regime Experiment							Coastal Regime Experiment			
Level of intervention												System-Level Intervention	
	Teacher-Level Intervention						Teacher Level Intervention						
			Student-Level Intervention							Student-Level Intervention			
Randomization Units*	108 schools randomized		11834 students randomized				532 teachers (classes) randomized			598 schools randomized			
Allocated	54 schools allocated to receive benchmarking letter	54 schools allocated to control (no letter)	3936 students allocated to lottery ticket for lesson completion treatment	3975 students allocated to encourage. messages on screen treatment	1962 students allocated to Plan and within household team-up treatment	1961 students allocated to control	177 teachers allocated to receive Administrative SMS	177 teachers allocated to receive encourage. emails	178 teachers allocated to control	149 schools allocated to receive lottery ticket for lesson completion and score treatment	150 schools allocated to receive Team-up with a peer treatment	149 schools allocated to receive schools Centralized Monitoring treatment	150 chools allocated to receive Decentralized Monitoring treatment
Received Treatment	97 teachers in 54 schools receive benchmarking letters**	101 teacher in 54 schools receive no letters**	3936 students receive lottery ticket for lesson completion	3975 students receive encourage. messages on screen	1962 students receive messages to plan and within household team-up	1961 students receive no messages	177 teachers receive administrative SMS	177 teachers receive encourage. emails	178 teachers do not receive SMS or emails	4025 students in 129 schools receive lottery ticket for lesson completion and score (if they enroll at least one student)***	4515 students in 126 schools receive messages to team-up with a peer (if they enroll at least one student)***	149 Schools are under Centralized Monitoring (119 schools enrolled at least one student)***	150 Schools are under Decentralized Monitoring (112 enrolled at least one student)***
A. Students number at the baseline assigned to the treatment	6890	8553	3936	3975	1962	1961	4693	4674	5031	4025	4515	3882	4125
B. Students number at endline knowldedge test	5541	6757	3884	3919	1947	1930	4429	4475	4810	3530	4047	3373	3612
Knowledge test Completion Rate in Percentage (A/B*100)	80.4	79	98.7	98.6	99.2	98.4	94.4	95.7	95.6	87.7	89.6	86.9	87.6
Analyzed for the time index (Students number)	6890	8553	3936	3975	1962	1961	4693	4674	5031	4025	4515	3882	4125
Analyzed for the knowledge test index (Students number)	5541	6757	3884	3919	1947	1930	4429	4475	4810	3530	4047	3373	3612

Note: *If the level of randomization was higher than the student, we used balanced randomization to boost statistical power. **In Zone 2 experiment (wave 1), 2 schools out of 108 randomized did not take-up the program (no information on time or knowledge index) and 4 more schools did not reach endline knowledge test (no information on knowledge index). ***In the Coastal experiment (wave 3), we randomized on the level of schools based on administrative information before students register on the platform (as we had to test decentralized monitoring). If none of the students enroll to the platform in school, the students from this school did not receive an email (student-level intervention) as they did not get through baseline. Anticipating that not all schools assigned will start to study, we specified Class Level Take-Up Index in a pre-analysis plan that test if the coverage (number of classes, number of students) substantially differs between treatments. We do not see that the coverage (number of classes, number of students) significantly differs between treatments neither on the 8th nor on the 11th week which additionally supports fidelity of randomization.

Figure S3: Example of a Benchmarking Letter

Estimado(a) docente(a)

Apreciamos mucho su esfuerzo y compromiso con el proyecto: **Develando Oportunidades de Vida**. Para garantizar la entrega de los cursos y ayudarlo a evaluar su desempeño, le proporcionamos este informe semanal, el cual consiste en lo siguiente:

(i) Gráfico de comparación de los resultados sobre el promedio de las lecciones completadas en sus clases vs. el promedio de lecciones completadas por los otros docentes con similar material de aprendizaje.

(ii) Gráfico de comparación de los resultados sobre el número de las lecciones completadas en sus clases vs. el máximo de lecciones completadas por los otros docentes con similar material de aprendizaje.

(iii) Cuadro con detalle del avance por clase.

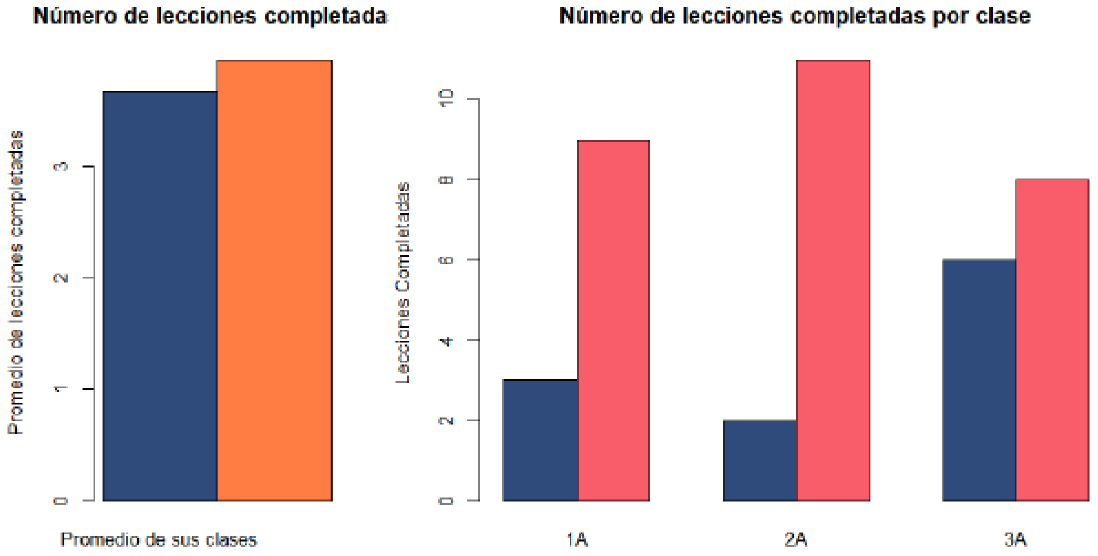

Figure S4 – Examples of Student-level Interventions in Wave 2

<table>
<tr>
<td>Treatment 1 -Control

No message is shown.</td>
<td>Treatment 2 - Encouragement

Each time the next group of screens shown at random:

Message 2.1

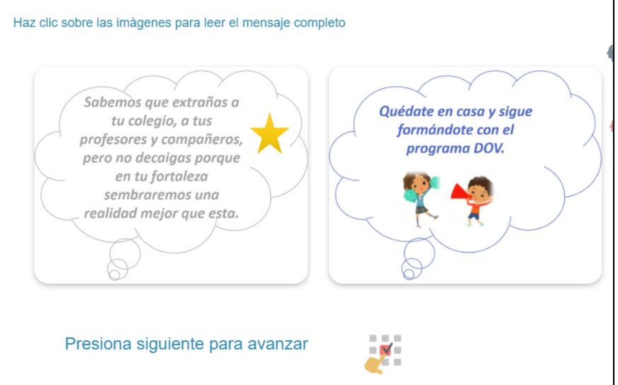

We know that you miss your school, your teachers and your classmates, but do not give up because in your strength we will sow a better reality than this.

Stay at home and continue training with the DOV Program.
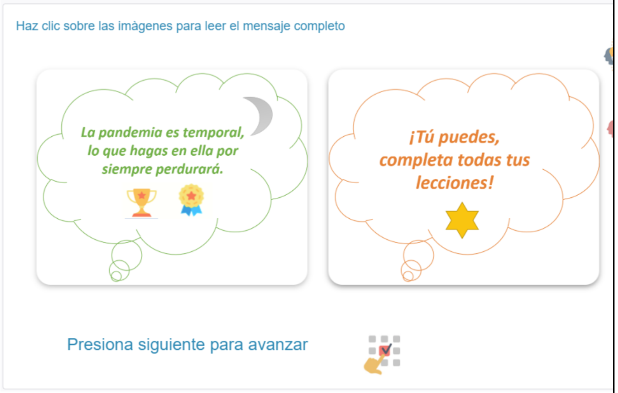

The pandemic is temporary, what you do in it will last forever.
You can, complete all your lessons!
Message 2.2</td>
</tr>
</table>

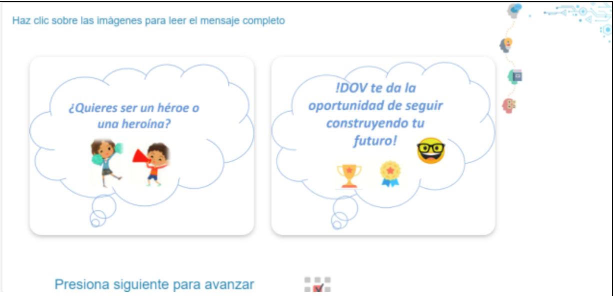

Do you want to be a hero or a heroine?
DOV gives you the opportunity to continue building your future!

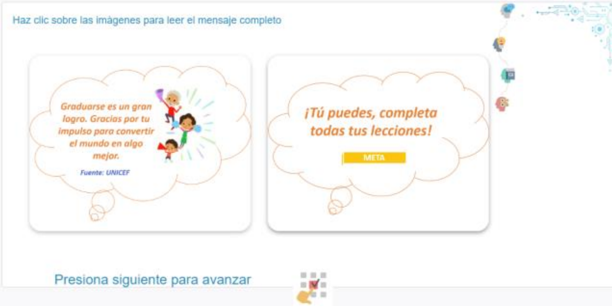

Graduating is a great achievement. Thank you for your drive to make the world a better place. Source: UNICEF.
You can, complete all your lessons!

Message 2.3

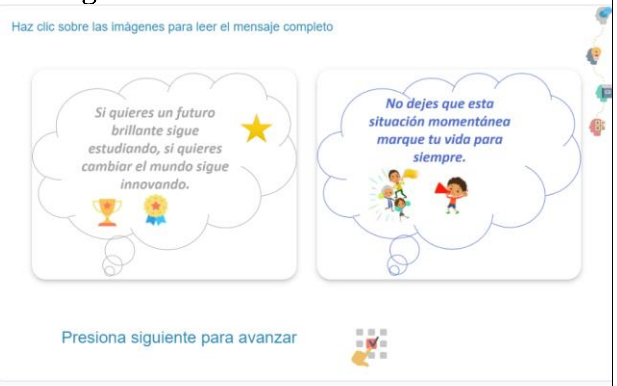

If you want a bright future keep studying, if you want to change the world keep innovating.
Do not let this momentary situation mark your life forever.

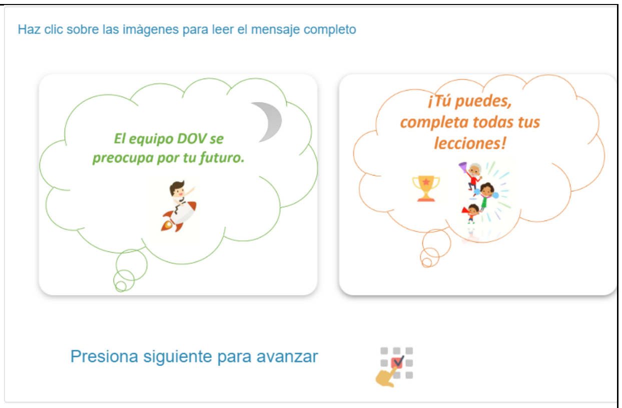

The DOV team cares about your future.
You can, complete all your lessons!

Message 2.4

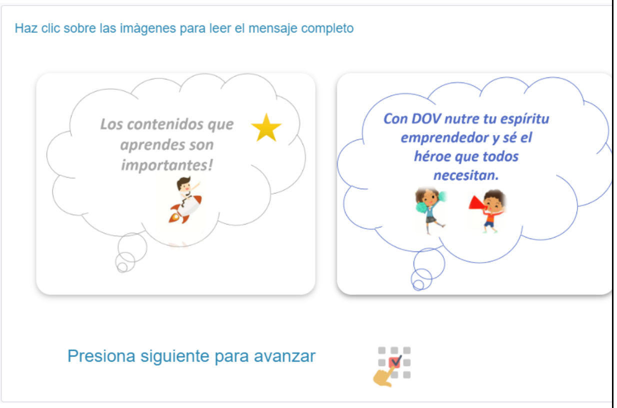

The contents you learn are important!
With DOV nourish your entrepreneurial spirit and be the hero that everyone needs.

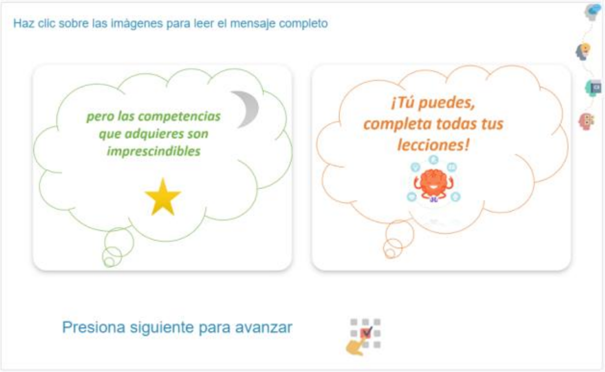

But the skills you acquire are essential.
You can, complete all your lessons!

Message 2.5

<table>
<tr>
<td></td>
<td>
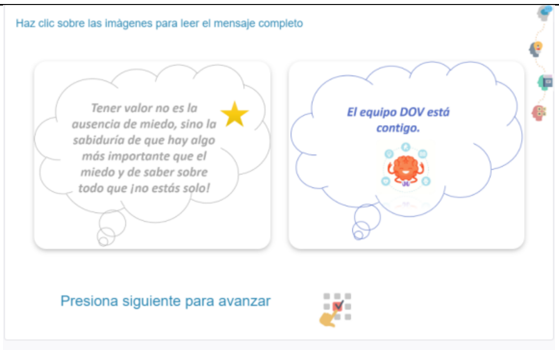

To have courage is not the absence of fear, but the wisdom that there is something more important than fear and to know above all that you are not alone!
The DOV team is with you.
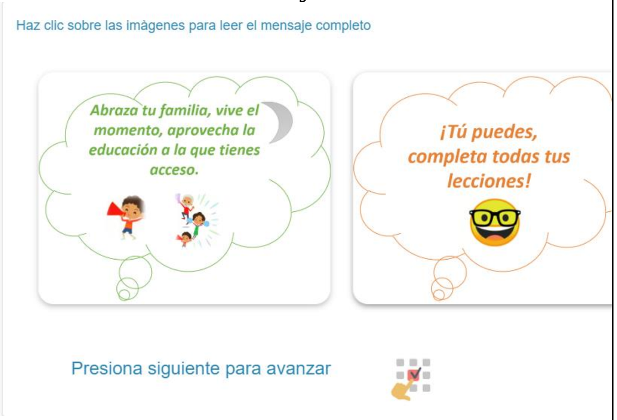

Hug your family, live in the moment, take advantage of the education you have access to.
You can, complete all your lessons!
</td>
</tr>
<tr>
<td>
Treatment 3 - Lottery Ticket
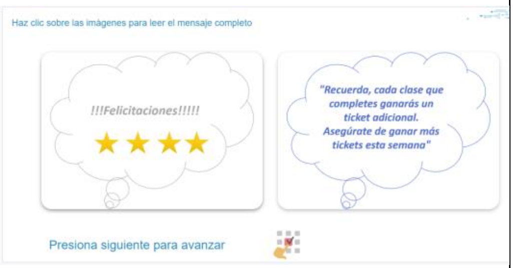

Congratulations!!!!!
"Remember, every class you complete will earn you an additional ticket. Make sure you win more tickets this week".
</td>
<td>
Treatment 4 - Team-up and make plans
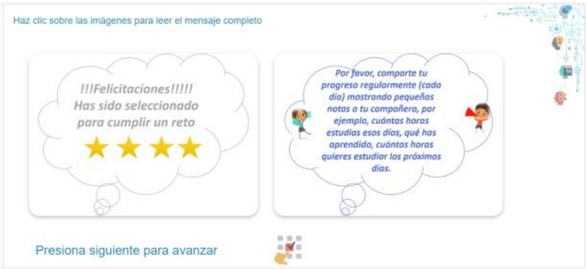

Congratulations!!!!!
You have been selected to meet a challenge
Please share your progress regularly (every day) by showing small notes to your partner, for example, how many hours you study on
</td>
</tr>
</table>

<table>
<tr>
<td>
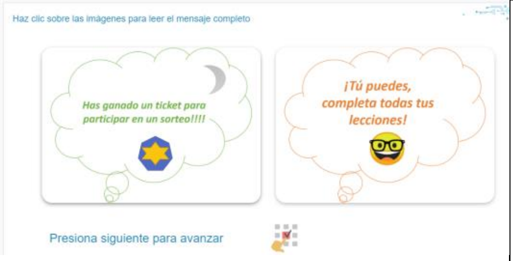

You have won a ticket to participate in a draw!!!!
You can, complete all your lessons!
</td>
<td>
those days, what you have learned, how many hours you want to study on the next few days.
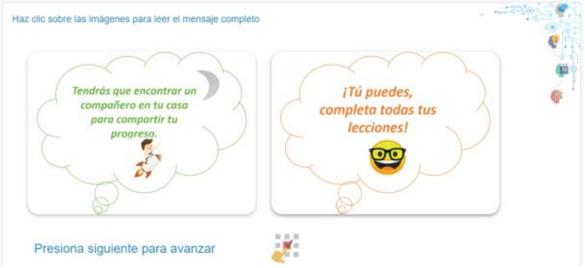

You will need to find a partner at home to share your progress.
You can, complete all your lessons!
</td>
</tr>
</table>

Figure S5: Examples of Teacher Encouragement Email

Email with Video

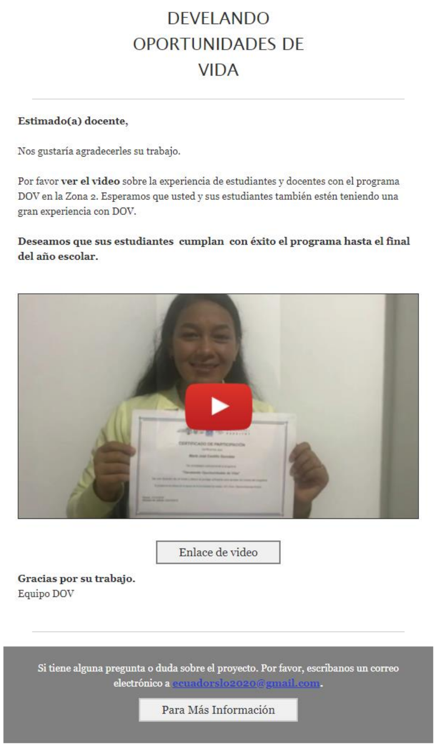

DEVELANDO OPORTUNIDADES DE VIDA

Estimado(a) docente,

Nos gustaría agradecerles su trabajo.

Por favor **ver el video** sobre la experiencia de estudiantes y docentes con el programa DOV en la Zona 2. Esperamos que usted y sus estudiantes también estén teniendo una gran experiencia con DOV.

Deseamos que sus estudiantes cumplan con éxito el programa hasta el final del año escolar.

Enlace de video

Gracias por su trabajo.
Equipo DOV

Si tiene alguna pregunta o duda sobre el proyecto. Por favor, escribanos un correo electrónico a ecuadorslo2020@gmail.com.

Para Más Información

Dear teacher,

We would like to thank you for your work.
Please see the video on the experience of students and teachers with the DOV Program in Zone 2. We hope that you and your students are also having a great experience with DOV.
We want your students to successfully complete the program until the end of the school year.

-Link to the video-

Thank you for your work.
DOV Team

If you have any questions or doubts about the project. Please email us at ecuadorslo2020@gmail.com.

For more information

Figure S6 – Example of the Centralized Management System and Self-Management System.

Centralized Management

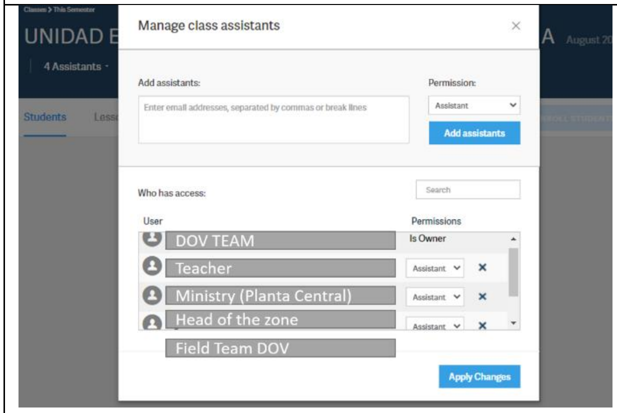

Self – Management

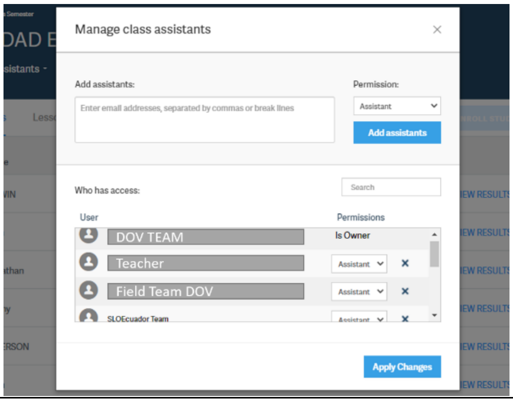

In Centralized Management, the ministry personnel (head of the educational zone, central office) is enrolled in each class monitoring dashboard, but in the self-management condition they are not. Thus, the ministry personnel do not see the information about performance of classes and students such as next:

List of enrolled students in class

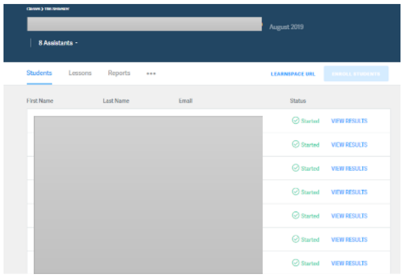

List of completed lesson in class

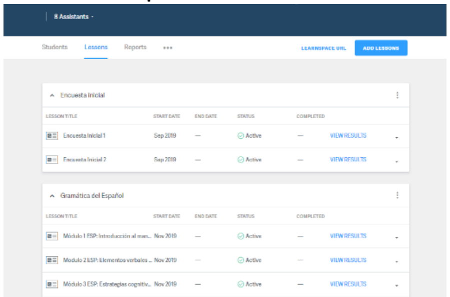

Student Results and progress

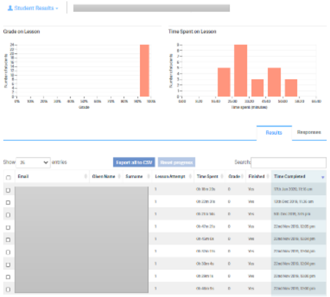

Lessons analytics

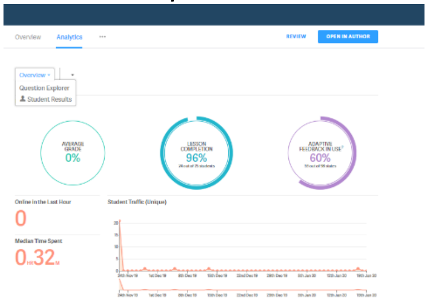

Individual student progress	Progress per student in class

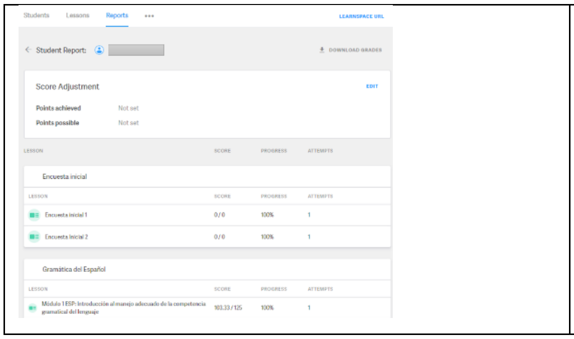

Students | Lessons | Reports | ••• LEARNSPACE URL

Student Report: DOWNLOAD GRADES

Score Adjustment EDIT

Points achieved Not set
Points possible Not set

LESSON	SCORE	PROGRESS	ATTEMPTS
**Encuesta inicial**			
Encuesta inicial 1	0 / 0	100%	1
Encuesta Inicial 2	0 / 0	100%	1
**Gramática del Español**			
Módulo 1 ESP: Introducción al manejo adecuado de la competencia gramatical del lenguaje	103.33 / 125	100%	1

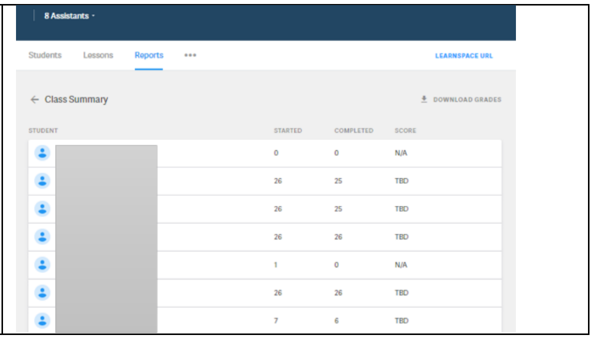

8 Assistants

Students | Lessons | Reports | ••• LEARNSPACE URL

Class Summary DOWNLOAD GRADES

STUDENT	STARTED	COMPLETED	SCORE
	0	0	N/A
	26	25	TBD
	26	25	TBD
	26	26	TBD
	1	0	N/A
	26	26	TBD
	7	6	TBD

With Centralized Management, Ministry officials get weekly monitoring reports from the system

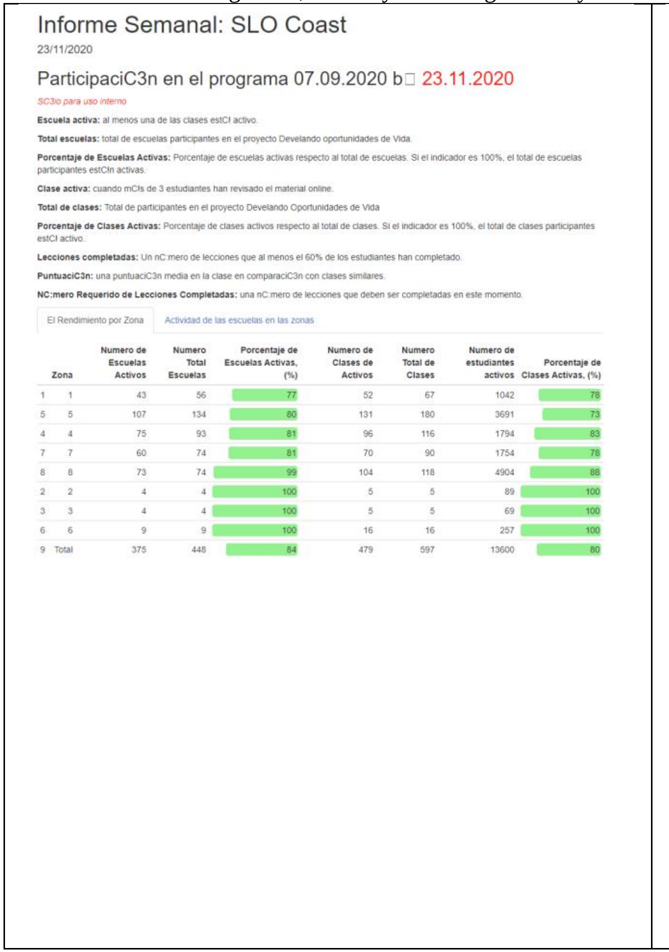

Informe Semanal: SLO Coast

23/11/2020

ParticipaciC3n en el programa 07.09.2020 b□ 23.11.2020

SC3lo para uso interno

Escuela activa: al menos una de las clases estC! activo.

Total escuelas: total de escuelas participantes en el proyecto Develando oportunidades de Vida.

Porcentaje de Escuelas Activas: Porcentaje de escuelas activas respecto al total de escuelas. Si el indicador es 100%, el total de escuelas participantes estC!n activas.

Clase activa: cuando mC!s de 3 estudiantes han revisado el material online.

Total de clases: Total de participantes en el proyecto Develando Oportunidades de Vida

Porcentaje de Clases Activas: Porcentaje de clases activos respecto al total de clases. Si el indicador es 100%, el total de clases participantes estC! activo.

Lecciones completadas: Un nC:mero de lecciones que al menos el 60% de los estudiantes han completado.

PuntuaciC3n: una puntuaciC3n media en la clase en comparaciC3n con clases similares.

NC:mero Requerido de Lecciones Completadas: una nC:mero de lecciones que deben ser completadas en este momento.

El Rendimiento por Zona | Actividad de las escuelas en las zonas

	Zona	Numero de Escuelas Activos	Numero Total Escuelas	Porcentaje de Escuelas Activas, (%)	Numero de Clases de Activos	Numero Total de Clases	Numero de estudiantes activos	Porcentaje de Clases Activas, (%)
1	1	43	56	77	52	67	1042	78
5	5	107	134	80	131	180	3691	73
4	4	75	93	81	96	116	1794	83
7	7	60	74	81	70	90	1754	78
8	8	73	74	99	104	118	4904	88
2	2	4	4	100	5	5	89	100
3	3	4	4	100	5	5	69	100
6	6	9	9	100	16	16	257	100
9	Total	375	448	84	479	597	13600	80

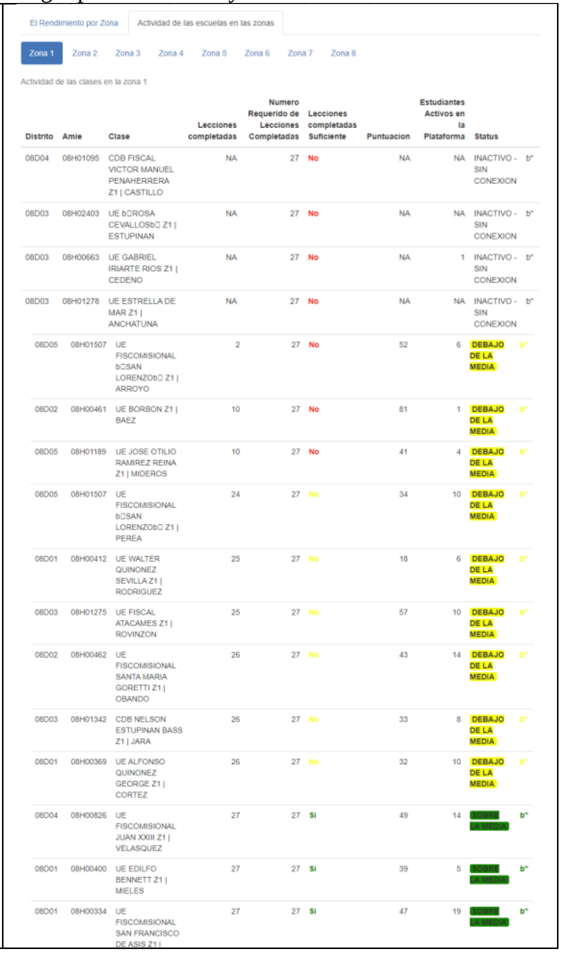

El Rendimiento por Zona | Actividad de las escuelas en las zonas

Zona 1 | Zona 2 | Zona 3 | Zona 4 | Zona 5 | Zona 6 | Zona 7 | Zona 8

Actividad de las clases en la zona 1

Distrito	Amie	Clase	Lecciones completadas	Numero Requerido de Lecciones Completadas	Lecciones completadas Suficiente	Puntuacion	Estudiantes Activos en la Plataforma	Status	
08D04	08H01095	CDB FISCAL VICTOR MANUEL PENAHERRERA Z1 | CASTILLO	NA	27	No	NA	NA	INACTIVO - SIN CONEXION	b"
08D03	08H02403	UE b□ROSA CEVALLOSb□ Z1 | ESTUPINAN	NA	27	No	NA	NA	INACTIVO - SIN CONEXION	b"
08D03	08H00663	UE GABRIEL IRIARTE RIOS Z1 | CEDENO	NA	27	No	NA	1	INACTIVO - SIN CONEXION	b"
08D03	08H01278	UE ESTRELLA DE MAR Z1 | ANCHATUNA	NA	27	No	NA	NA	INACTIVO - SIN CONEXION	b"
08D05	08H01507	UE FISCOMISIONAL b□SAN LORENZOb□ Z1 | ARROYO	2	27	No	52	6	DEBAJO DE LA MEDIA	b"
08D02	08H00461	UE BORBON Z1 | BAEZ	10	27	No	81	1	DEBAJO DE LA MEDIA	b"
08D05	08H01189	UE JOSE OTILIO RAMIREZ REINA Z1 | MIDEROS	10	27	No	41	4	DEBAJO DE LA MEDIA	b"
08D05	08H01507	UE FISCOMISIONAL b□SAN LORENZOb□ Z1 | PEREA	24	27	No	34	10	DEBAJO DE LA MEDIA	b"
08D01	08H00412	UE WALTER QUINONEZ SEVILLA Z1 | RODRIGUEZ	25	27	No	18	6	DEBAJO DE LA MEDIA	b"
08D03	08H01275	UE FISCAL ATACAMES Z1 | ROVINZON	25	27	No	57	10	DEBAJO DE LA MEDIA	b"
08D02	08H00462	UE FISCOMISIONAL SANTA MARIA GORETTI Z1 | OBANDO	26	27	No	43	14	DEBAJO DE LA MEDIA	b"
08D03	08H01342	CDB NELSON ESTUPINAN BASS Z1 | JARA	26	27	No	33	8	DEBAJO DE LA MEDIA	b"
08D01	08H00369	UE ALFONSO QUINONEZ GEORGE Z1 | CORTEZ	26	27	No	32	10	DEBAJO DE LA MEDIA	b"
08D04	08H00826	UE FISCOMISIONAL JUAN XXIII Z1 | VELASQUEZ	27	27	Si	49	14	SOBRE LA MEDIA	b"
08D01	08H00400	UE EDILFO BENNETT Z1 | MIELES	27	27	Si	39	5	SOBRE LA MEDIA	b"
08D01	08H00334	UE FISCOMISIONAL SAN FRANCISCO DE ASIS Z1 |	27	27	Si	47	19	SOBRE LA MEDIA	b"

Figure S7 – Examples of Messages to Students in wave 3 (Coastal Regime) Lottery Treatment

Si tiene alguna pregunta o duda sobre el proyecto. Por favor, escribanos un correo electrónico a ecuadorslo2020@gmail.com.
Para más información

Message translated:

Subject: Hello [student_name]! Good news! Come and participate! - DOV Project 😉

Hello [student_name]!,

Congratulations! The DOV program has selected you to participate in a lottery:

The more lessons you study and the better your score on the platform, the more likely you are to receive the monetary prize each week.

Remember:

- We will make weekly draws. Your chances of winning depend on your weekly progress on the platform.

- For every lesson you complete and the better your score, the more chances you have of winning the monetary prize. You can retake a lesson if you want to get a better grade - your last attempt will be counted. The goal is for you to learn as much as you can!

- Make sure you win this week and enjoy learning your lessons.

Accept the challenge!

You can complete all your lessons and learn as much as you can from the lessons!

Yours sincerely,

DOV Team

If you have any questions or doubts about the project. Please email us at ecuadorslo2020@gmail.com.

For more information

Placebo reminder in lottery treatment:

Asunto: ¡Hola [nombre_estudiante]! Proyecto DOV ☺

DEVELANDO
OPORTUNIDADES DE VIDA

Hola [nombre_estudiante]!,

¡Puedes completar todas tus lecciones y aprender todo lo que puedas de las lecciones!

Atentamente,
Equipo DOV

Si tiene alguna pregunta o duda sobre el proyecto. Por favor, escríbanos un correo electrónico a ecuadorslo2020@gmail.com.
Para más información

Subject: Hello [student_name]! DOV Project ☺

Hello [student_name]!

You can complete all your lessons and learn as much as you can from the lessons!

Yours sincerely,

DOV Team

If you have any questions or doubts about the project. Please email us at ecuadorslo2020@gmail.com.

For more information

Asunto: ¡Hola [nombre_estudiante]! ¡Buenas noticias! ¡Anímate a participar! - Proyecto DOV

DEVELANDO
OPORTUNIDADES DE VIDA

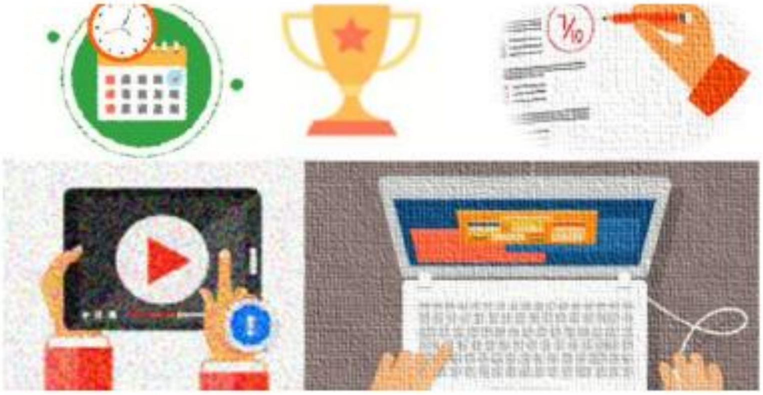

Hola [nombre_estudiante]!,

¡Felicidades! El programa DOV te ha seleccionado para participar en una lotería:

Cuantas más lecciones estudies y mejor sea tu puntuación en la plataforma, más probabilidades tendrás de recibir el premio monetario cada semana.

Recuerda:

- Haremos sorteos semanales. Tus oportunidades de ganar dependen de tu progreso semanal en la plataforma.
- Por cada lección que completes y mientras mejor sea tu puntaje, más oportunidades tendrás de ganar el premio monetario. Puedes volver a hacer una lección si quieres obtener una mejor nota - tu último intento será contado. ¡El objetivo es que aprendas todo lo que puedas!
- Asegúrate de ganar esta semana y disfruta aprendiendo tus lecciones.

!Acepten el desafío!
¡Puedes completar todas tus lecciones y aprender todo lo que puedas de las lecciones!

Atentamente,
Equipo DOV

Figure S8: Examples of Messages to Students in wave 3 (Coastal Regime) Team-Up Treatment

Asunto: ¡Hola [nombre_estudiante]! ¿Aceptas el reto? ¡Anímate a participar! - Proyecto DOV

DEVELANDO
OPORTUNIDADES DE VIDA

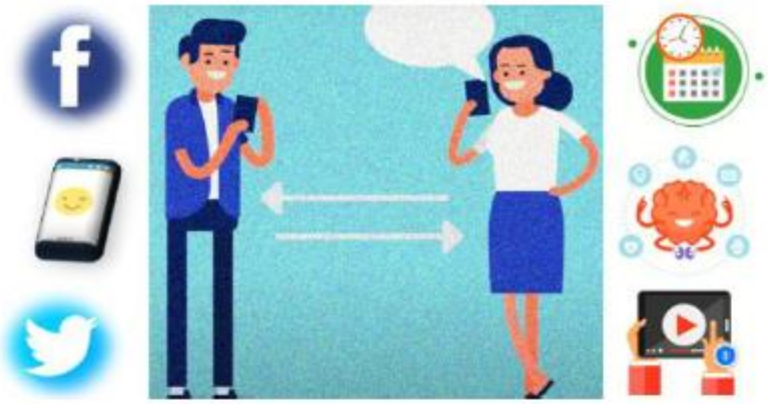

Hola [nombre_estudiante]! ,

¡Felicidades! El programa DOV te ha seleccionado para que cumplas un reto:

¡Únete a un(a) compañero(a) de clase a distancia para estudiar los materiales del proyecto DOV y logra mejores resultados en tus estudios!

Recuerda, si estudian juntos pueden obtener mejores resultados en el curso.

¿Qué debo hacer?

- Comuníquense por WhatsApp, teléfono, redes sociales. Evita la interacción en persona.
- Compartan sus progresos regularmente (¡cada día!), por ejemplo, cuántas horas estudian diario, qué han aprendido, cuántas horas quieren estudiar en los próximos días.
- Ayúdense mutuamente e intenten discutir el material que encuentren especialmente interesante o desafiante.

!Acepten el desafío!
¡Juntos pueden completar todas tus lecciones y lograr los mejores resultados de aprendizaje!

Atentamente,
Equipo DOV

Si tiene alguna pregunta o duda sobre el proyecto. Por favor, escríbanos un correo electrónico a ecuadorslo2020@gmail.com.
Para más información

Subject: Hello [student_name]! Will you accept the challenge? Come and participate! - DOV Project

Message translated:

Hello [student_name]!,

Congratulations! The DOV Program has selected you to meet a challenge:

Join a classmate at a distance to study the DOV project materials and achieve better results in your studies!

Remember, if you study together you can get better results on the course.

What should I do?

- Communicate by WhatsApp, phone, social networks. Avoid in-person interaction.

- Share your progress regularly (every day!), for example, how many hours you study each day, what you have learned, how many hours you want to study in the next few days.

- Help each other and try to discuss the material you find particularly interesting or challenging.

Accept the challenge!

Together you can complete all your lessons and achieve the best learning results!

Yours sincerely,

DOV Team

If you have any questions or doubts about the project. Please email us at ecuadorslo2020@gmail.com.

For more information

Placebo reminder in team-up treatment :

DEVELANDO
OPORTUNIDADES DE VIDA

Hola [nombre_estudiante]!,

¡Puedes completar todas tus lecciones y lograr los mejores resultados de aprendizaje!

Atentamente,
Equipo DOV

Si tiene alguna pregunta o duda sobre el proyecto. Por favor, escríbanos un correo electrónico a ecuadorslo2020@gmail.com.
Para más información

Asunto: ¡Hola [nombre_estudiante]! Proyecto DOV ☺

Hello [student_name]!

You can complete all your lessons and achieve the best learning results!

Yours sincerely,

DOV Team

If you have any questions or doubts about the project. Please email us at ecuadorslo2020@gmail. com.

For more information

Subject: Hello [student_name]! DOV Project ☺

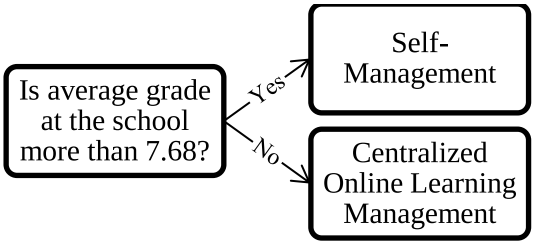

Figure S9: Policy Tree. Self-Management Vs. Centralized Management Group. The outcome of interest: overall knowledge index

SI References

1. A. Belloni, V. Chernozhukov, C. Hansen, Inference on Treatment Effects after Selection among High-Dimensional Controls, *Review of Economic Studies* 81(2): 608-650, 2014.
2. D. Viviano, K. Wüthrich and P. Niehaus (When) should you adjust inferences for multiple hypothesis testing? UC San Diego Working Paper. 2022.
3. P. Bergman, M.J. Hill, The effects of making performance information public: Regression discontinuity evidence from Los Angeles teachers. Economics of Education Review, 66, pp.104-113, 2018.
4. S. Athey, S.Wager. Policy learning with observational data. *Econometrica*, 89(1): 133-161, 2021.
5. D. Hardt, M. Nagler, J. Rincke, Can peer mentoring improve online teaching effectiveness? an RCT during the Covid-19 pandemic. 2020.
